# The Effect of Surface Roughness on Supersonic Nozzle Flow and Electron Dispersion at Low Pressure Conditions

**DOI:** 10.3390/s25134204

**Published:** 2025-07-05

**Authors:** Pavla Šabacká, Jiří Maxa, Robert Bayer, Tomáš Binar, Petr Bača

**Affiliations:** 1 Faculty of Electrical Engineering and Communication, Brno University of Technology, Technická 10, 616 00 Brno, Czech Republic; 2 Institute of Scientific Instruments of the CAS, Královopolská 147, 612 64 Brno, Czech Republic; 3 Faculty of AgriSciences, Mendel University in Brno, Zemědělská 1665/1, 613 00 Brno, Czech Republic

**Keywords:** Ansys Fluent, aperture, CFD, differentially pumped chamber, ESEM, low pressure, nozzle, roughness, shock wave

## Abstract

This study investigates supersonic flow within a nozzle under low-pressure conditions at the continuum mechanics boundary. This phenomenon is commonly encountered in applications such as the differentially pumped chamber of an Environmental Scanning Electron Microscope (ESEM), which employs an aperture to separate two regions with a great pressure gradient. The nozzle geometry and flow control in this region can significantly influence the scattering and loss of the primary electron beam traversing the differentially pumped chamber and aperture. To this end, an experimental chamber was designed to explore aspects of this low-pressure regime, characterized by a varying ratio of inertial to viscous forces. The initial experimental results obtained using pressure sensors from the fabricated experimental chamber were utilized to refine the Ansys Fluent simulation setup, and in this combined approach, initial analyses of supersonic flow and shock waves in low-pressure environments were conducted. The refined Ansys Fluent system demonstrated a very good correspondence with the experimental findings. Subsequently, an analysis of the influence of surface roughness on the resulting flow behavior in low-pressure conditions was performed on this refined model using the refined CFD model. Based on the obtained results, a comparison of the influence of nozzle roughness on the resulting electron beam scattering was conducted for selected low-pressure variants relevant to the operational conditions of the Environmental Scanning Electron Microscope (ESEM). The influence of roughness at elevated working pressures within the ESEM operating regime on reduced electron beam scattering has been demonstrated. At lower pressure values within the ESEM operating regime, this influence is significantly diminished.

## 1. Introduction

Research in the field of Environmental Scanning Electron Microscopy (ESEM) is being conducted at the Institute of Scientific Instruments of the Academy of Sciences of the Czech Republic (ISI CAS). ESEM enables the observation of non-conductive [1,2], semiconductive [3], and native samples [4,5,6] without causing damage, which distinguishes it from conventional electron microscopes. The research encompasses the area of vacuum chamber pumping, particularly the pumping of a differentially pumped chamber, in which the Department of Electrotechnology at Brno University of Technology, Faculty of Electrical Engineering and Communication, also participates. This research utilizes a modern methodology that combines physical theory, Computational Fluid Dynamics (CFD) analyses, and experimental measurements, which leverages the combination of CFD analyses for precise sensor placement to minimize measurement errors.

**Figure 1:**
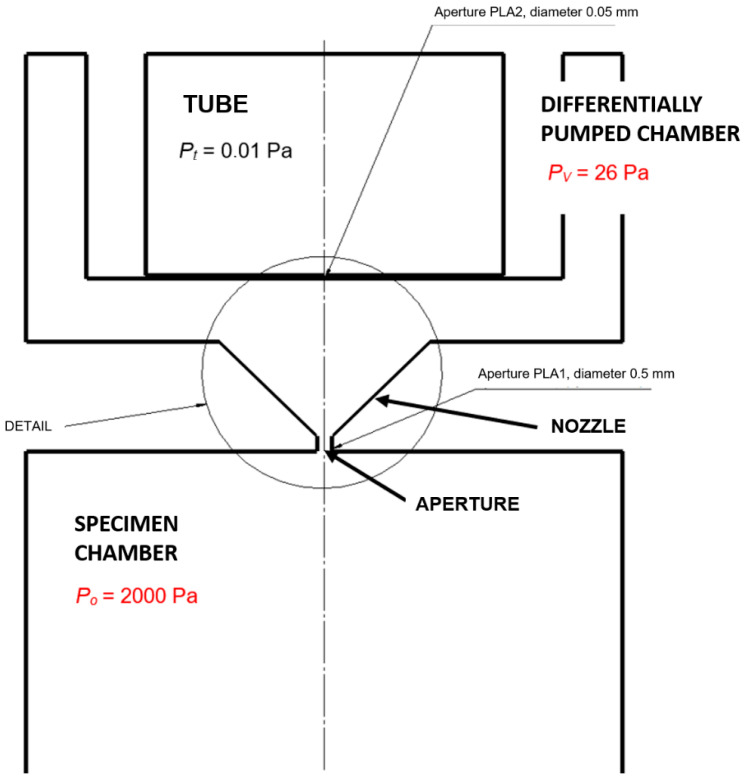
Environmental Scanning Electron Microscope (ESEM)—chamber scheme.

The differentially pumped chamber and the specimen chamber are separated by a small-diameter aperture fitted with a nozzle (Figure 1), which induces the formation of critical flow in a supersonic flow regime, culminating in a shock wave. The pressure distribution, shock wave characteristics, and overall flow behavior within the primary electron beam path traversing the differentially pumped chamber significantly impact the resulting image quality.

Dr. Danilatos is considered a pioneer in differential pumping technology, having dedicated extensive research to this field [7]. His long-term investigations have focused on the influence of nozzle thickness, in the absence of an aperture, on the resultant electron beam transmission [8,9]. Furthermore, he has provided fundamental insights into the density and pressure variations observed in gas flow through small apertures separating chambers with significant pressure differentials. Utilizing a ThermoFisher electron microscope, Dr. Danilatos has examined the impact of aperture size and controlled back pressure on gas flow rates [10,11,12].

**Figure 2:**
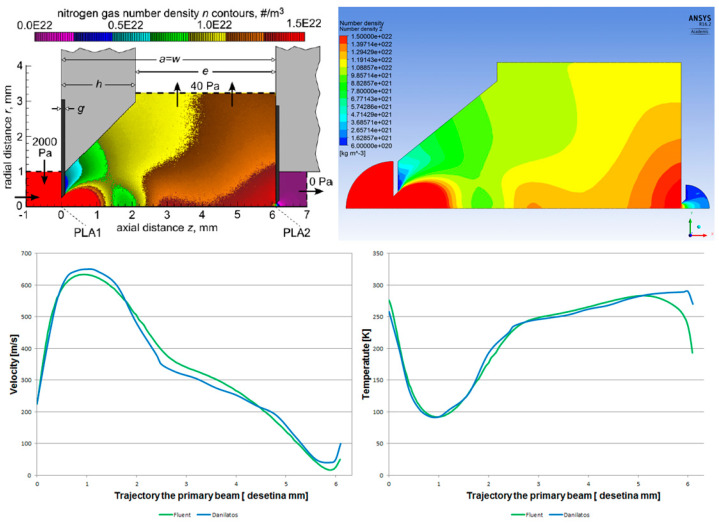
Density distribution obtained by Dr. Danilatos using the Monte Carlo method (**left**) and the continuum mechanics method (**right**).

Dr. Danilatos employs a refined Monte Carlo system for his computations, which utilizes statistical methodologies, thereby allowing for potential system extension. In the context of the present article, all CFD analyses were conducted using the Ansys Fluent system, which operates on the principles of continuum mechanics, as described by the Navier–Stokes equations. Our team has previously performed a comparative analysis of these two methodologies, yielding identical results, as shown in Figure 2 [13].

Building upon prior experience and the established tuning of the Ansys Fluent system for this specific computational application, the density-based solver was employed, utilizing an implicit scheme with second- and third-order discretization and the SST-omega turbulence model. The specific refinement of the CFD analysis for the case presented herein will be detailed subsequently.

CFD analyses serve as a crucial tool for the description of supersonic flow at low pressures, particularly in the analysis of shock wave distribution and abrupt changes in pressure, temperature, and density. CFD analyses enable a detailed characterization of shock wave formation and propagation, including the area where oblique shock waves interact with object surfaces, in this case, the nozzle surface. Consequently, CFD analyses constitute a pivotal instrument in the design of supersonic nozzles employed in diverse applications, such as rocket engines. Simulations facilitate the analysis of flow within the nozzle and enable the modification of its geometry to achieve desired output flow characteristics. In this contribution, the minimization of pressure along the flow axis is paramount to reduce electron beam dispersion.

This study focuses on the analysis of flow characteristic differences arising from nozzle surface irregularities and their interaction with the boundary layer, and subsequently, on the properties of the resultant shock waves. These shock waves exert a critical influence on the scattering of the primary electron beam during its passage through a differentially pumped chamber, thereby affecting the quality of the final image. Within this domain, a significant alteration in the ratio between inertial and viscous forces occurs. This alteration has a substantial impact on the formation and intensity of shock waves. At low pressures, a discernible reduction in inertial forces is observed, attributable to decreased density. Conversely, viscous forces remain essentially constant down to a pressure of approximately 133 Pa, exhibiting negligible variation with further pressure reduction. This research is conducted utilizing a universal experimental chamber.

Subsequently, an analysis was conducted to ascertain the impact of nozzle surface roughness on the scattering of the passing electron beam. Beam dispersion constitutes a key parameter influencing the resultant image sharpness within an ESEM. The beam dispersion for individual configurations was evaluated based on the acquired data [14].

To acquire the aforementioned results, the Ansys Fluent system was refined against experimentally obtained data, achieving an agreement ranging from 0.21% to 18%. This level of agreement is considered acceptable, as results within a 20% deviation are deemed satisfactory. Building upon this refined model, initial analyses were conducted on the geometry of the experimental chamber at pressure variants of 10,000 Pa and 2000 Pa, both with a pressure ratio of 10:1. These analyses encompassed roughness variants including No roughness variant, 1.6, 3.2, and 6.3, unequivocally demonstrating the influence of roughness on the flow characteristics. Subsequently, analogous analyses were performed, but for the actual geometry of the AQUASEM II ESEM, evaluating beam dispersion for two operational pressure regimes of this microscope. A discernible impact of roughness was identified at the upper limit of the operational regime, manifesting in one critical location with a beam dispersion of approximately Mk = 0.05. At lower operational pressures, this influence diminished.

## 2. Methodology

### 2.1. Experimental Chamber

This research, conducted at the ISI CAS in collaboration with the Department of Electrical Engineering and Electronics, Faculty of Electrical Engineering and Communication, Brno University of Technology, investigates the phenomena associated with the pumping of vacuum chambers characterized by great pressure gradients, specifically a ratio of approximately *p*_0_:*p_v_* = 10:1 (Figure 1). The presented paper forms a component of this broader research endeavor, focusing on the study of supersonic flow within apertures and nozzles at low pressures, occurring at the continuum mechanics boundary and rarefied gas dynamics, encountered during the pumping of the differentially pumped chamber. This research is particularly significant due to the operational environment of differentially pumped chambers, which operate at low pressures where the ratio of inertial to viscous forces deviates significantly from typical atmospheric conditions.

**Figure 3:**
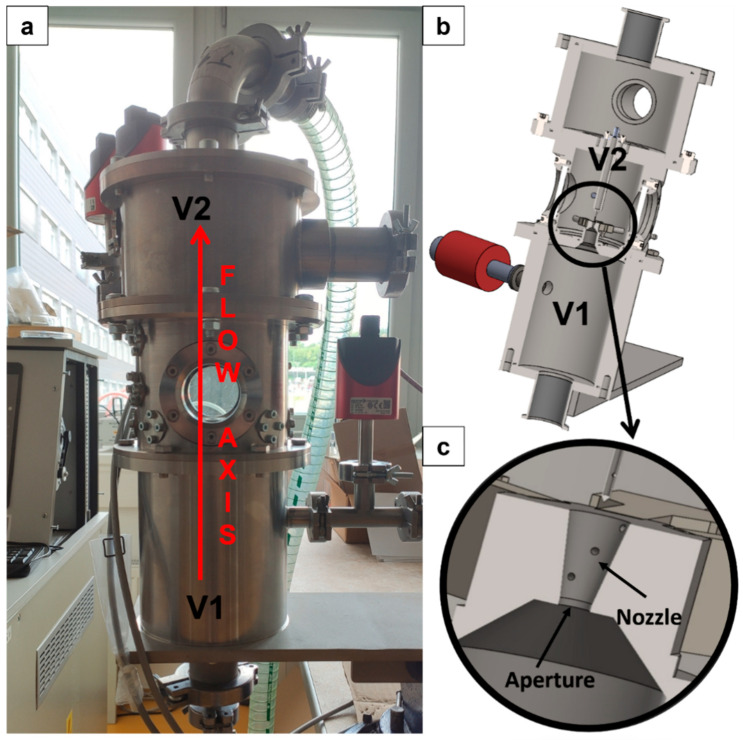
Real experimental chamber (**a**) in comparison with 3D model of experimental chamber (**b**) with detail of aperture fitted with nozzle (**c**).

The geometry of the chamber, and consequently, the way the flow and shock wave morphology are controlled within it, exerts a significant influence on the dispersion of the transmitted primary beam. For this reason, the experimental chamber was designed to investigate supersonic flow phenomena under conditions of substantial pressure differentials between two chambers separated by an aperture and a nozzle (Figure 3c). This chamber simulates the flow dynamics between a specimen chamber (designated V1) and a differentially pumped chamber (designated V2) (Figure 3a). Figure 3 further provides a comparative visualization of the fabricated experimental chamber and its corresponding 3D volumetric model (Figure 3b).

**Figure 4:**
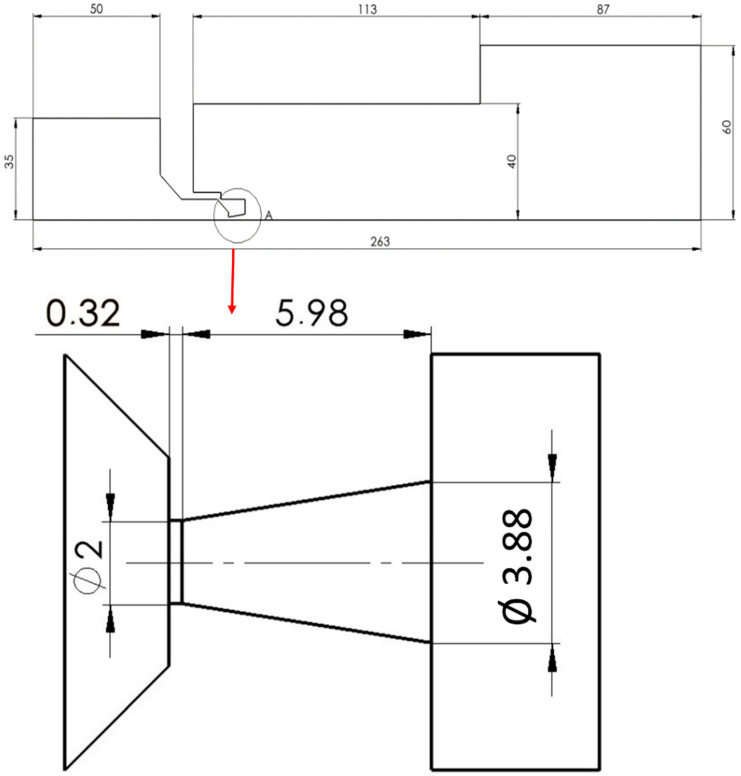
2D axisymmetric model rotated 90°, including the model’s dimensions.

As will be detailed subsequently, the entire research is conducted through a combination of physical theory, predominantly the theory of one-dimensional isentropic flow, CFD analyses, and experimental measurement [15,16]. Consequently, preliminary CFD analyses were performed prior to manufacturing, informing the design of the experimental chamber and the selection of high-precision pressure sensors to minimize measurement error. Following manufacturing, the actual dimensions of the chamber were experimentally validated using a TESCAN VEGA3 electron microscope (TESCAN GROUP, a.s., Brno, Czech republic). These dimensions were then integrated into the CFD model to ensure maximum accuracy in subsequent CFD analyses (Figure 4).

The passage of flow through the aperture results in a specific phenomenon known as nozzle choking. This manifests as the formation of critical flow, followed by the generation of supersonic flow characterized by a low-pressure region terminated by a shock wave of a certain type [17,18,19]. This region behind the aperture arises from the constraint that only a quantity of gas transiting at sonic velocity can pass through the aperture. The supersonic flow regime concludes with a shock wave of a defined type, indicating a region of increased gas density [20]. The described flow through the aperture is governed by relationships between state variables such as pressure, temperature, density, velocity, and Mach number, as articulated by the theory of one-dimensional isentropic flow. These state variables and their interrelationships are described by the subsequent equations (Equations (1)–(6)), which are meticulously described and rigorously derived in the publication Fundamentals of Engineering Thermodynamics by Moran et al. [19,21]. Drawing upon the theory of one-dimensional flow (Equations (1)–(6)), the computational nozzle cross-section (Figure 4) was determined, the derivation of which was meticulously detailed in [14].

(1) $$\frac{v_{v}}{v_{kr}}={\left[\frac{\left(\varkappa +1\right)M^{2}}{2+\left(\varkappa -1\right)M^{2}}\right]}^{\frac{1}{2}}$$

(2) $$\frac{a_{v}}{a_{0}}={\left[\frac{2}{2+\left(\varkappa -1\right)M^{2}}\right]}^{\frac{1}{2}}$$

(3) $$\frac{T_{v}}{T_{0}}=\frac{2}{2+\left(\varkappa -1\right)M^{2}}$$

(4) $$\frac{p_{v}}{p_{0}}={\left[\frac{2}{2+\left(\varkappa -1\right)M^{2}}\right]}^{\frac{\varkappa }{\varkappa -1}}$$

(5) $$\frac{\rho_{v}}{\rho_{0}}={\left[\frac{2}{2+\left(\varkappa -1\right)M^{2}}\right]}^{\frac{1}{\varkappa -1}}$$

(6) $$\frac{\rho_{v}}{\rho_{kr}}=\frac{A_{kr}}{A}=M{\left[\frac{\varkappa +1}{2+\left(\varkappa -1\right)M^{2}}\right]}^{\frac{1\varkappa +1}{2\varkappa -1}}$$

where *p*_0_ [Pa] is input pressure, *p_v_* [Pa] is output pressure, *T*_0_ [K] is input temperature, *T_v_* [K] is output temperature, *a*_0_ [m·s^−1^] is input speed of sound, *a_v_* [m·s^−1^] is output speed of sound, *v_v_* [m·s^−1^] is output velocity, *v_kr_* [m·s^−1^] is critical velocity, *ρ*_0_ [kg·m^−3^] is input density, *ρ_v_* [kg·m^−3^] is output density, *M* [-] is Mach number, ϰ [-] is Poisson constant = 1.4, *A* [m^2^] is computational cross-section, and *A_kr_* [m^2^] is critical cross-section. *a*_0_ is the speed of sound within the given medium in the chamber upstream of the aperture, where the velocity is zero.

(7) $$a_{0}=\sqrt{\varkappa RT}$$

where *R* [-] is universal gas constant equal to 287, and *T* [K] is temperature of given medium (temperature in chamber V1 is 24 °C).

Nitrogen was employed as the working fluid in all experiments. In the CFD analyses, the physical properties of this gas were sourced from the NIST database, treated as a real gas rather than an ideal gas, to ensure the simulation results closely approximate real-world conditions [22].

**Figure 5:**
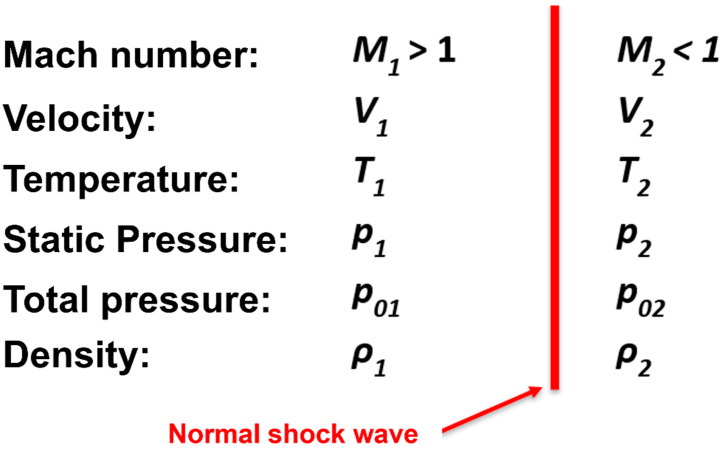
Changes in state variables across a normal shock wave.

Subsequent analyses, employing one-dimensional isentropic flow principles, encompass the evaluation of flow across a normal shock wave. Following the execution of CFD analyses, the results were validated utilizing the second segment of one-dimensional isentropic flow theory (Equations (8)–(14)) [21], which addresses the variations in state variables during the traversal of a normal shock wave (Figure 5). The purpose of this theoretical application is to corroborate the findings derived from the CFD analyses.

(8) $$M_{1n}=M_{1}\sin\alpha_{s}$$

(9) $$M_{2}^{2}=\frac{2+\left(\varkappa -1\right)M_{1n}^{2}}{2\varkappa M_{1n}^{2}-\left(\varkappa -1\right)}$$

(10) $$\frac{T_{2}}{T_{1}}=1+\frac{2\left(\varkappa -1\right)}{{\left(\varkappa +1\right)}^{2}}\cdot \frac{1+\varkappa M_{1n}^{2}}{M_{1n}^{2}}\cdot \left(M_{1n}^{2}-1\right)$$

(11) $$\frac{\rho_{2}}{\rho_{1}}=\frac{V_{1}}{V_{2}}=\frac{\left(\varkappa +1\right)M_{1n}^{2}}{2+\left(\varkappa -1\right)M_{1n}^{2}}$$

(12) $$\frac{p_{02}}{p_{01}}={\left[1+\frac{2\varkappa }{\varkappa +1}\left(M_{1n}^{2}-1\right)\right]}^{-\frac{1}{\varkappa -1}}{\left[\frac{\left(\varkappa +1\right)M_{1n}^{2}}{2+\left(\varkappa -1\right)M_{1n}^{2}}\right]}^{\frac{\varkappa }{\varkappa -1}}$$

(13) $$\frac{p_{02}}{p_{1}}={\left[1+\frac{2\varkappa }{\varkappa +1}\left(M_{1n}^{2}-1\right)\right]}^{-\frac{1}{\varkappa -1}}{\left[\frac{\varkappa +1}{2}M_{1n}^{2}\right]}^{\frac{\varkappa }{\varkappa -1}}$$

(14) $$\frac{p_{2}}{p_{1}}=1+\frac{2\varkappa }{\varkappa +1}\left(M_{1n}^{2}-1\right)$$

where *M*_1*n*_ [-] is normal component of the Mach number, *M*_2_ [-] is Mach number behind the normal shock wave, *T*_2_ [K] is temperature behind the shock wave, *T*_1_ [K] is temperature in front of the shock wave, *p*_2_ [Pa] is static pressure behind the shock wave, *p*_1_ [Pa] is static pressure in front of the shock wave, *ρ*_2_ [kg·m^−3^] is density behind the shock wave, *ρ*_1_ [kg·m^−3^] is density in front of the shock wave, *p*_02_ [Pa] is total pressure behind the shock wave, and *p*_01_ [Pa] is total pressure in front of the shock wave.

The aforementioned relations from the first part of the one-dimensional isentropic flow theory (Equations (1)–(6)) served as the basis for the initial computational design of the nozzle, on which CFD analyses were performed (specified for a pressure ratio of 2000:70). Subsequently, a comparison with results obtained using the one-dimensional isentropic flow theory was conducted.

### 2.2. Ansys Fluent Settings

The given pressure drop between chambers V1 and V2, separated by a small orifice, and the theory of one-dimensional isentropic flow predict substantial pressure and temperature gradients [23,24,25]. Two solvers were considered and tested: a density-based solver and a pressure-based solver [26].

Following the comparison of the aforementioned solvers, a density-based solver was employed. This solver concurrently addresses the governing equations for continuity, momentum, energy, and species transport, while processing additional scalar equations sequentially. The complex flow dynamics within the nozzle necessitate an implicit linearization approach for solving the coupled equations. A coupled implicit approach, which solves all variables simultaneously across cells, demonstrated stability and robustness in handling the intricate supersonic flow and steep pressure gradients within the experimental chamber.

Furthermore, the Advection Upstream Splitting Method (AUSM) was employed to discretize the convective and compressive fluxes. This scheme utilizes the eigenvalues of Jacobian flux matrices. The AUSM scheme offers several advantages, including the following:

- Precise capturing of shock and contact discontinuities;
- Solution possibilities for entropy preservation;
- Suppression of the carbuncle phenomenon, a common numerical instability associated with low-dissipation shock-capturing schemes;
- Robust accuracy and convergence across a broad range of Mach numbers.

Notably, the efficacy of the method does not rely on explicit eigenvector information, rendering it suitable for systems with intricate, undefined eigenstructures, such as those encountered in two-fluid multiphase flow models [27].

At the core of the Ansys Fluent system are the Reynolds-averaged Navier–Stokes (RANS) equations, which are fundamentally based on the Reynolds decomposition. This decomposition separates each instantaneous quantity (e.g., velocity or pressure) into its time-averaged component and a fluctuating component. The fundamental formulation of the RANS equations begins with the following continuity equation:

(15) $$\frac{\partial {\bar{u}}_{i}}{\partial x_{i}}=0$$

where ${\bar{u}}_{i}$ is the *i*-th component of the mean velocity, and $x_{i}$ is the *i*-th coordinate.

This equation simply states that the mean flow must also adhere to the principle of mass conservation; thus, for incompressible flow, the divergence of the mean velocity field is zero. The momentum equation is subsequently defined as follows:

(16) $$\frac{\partial \left(\varrho {\bar{u}}_{i}\right)}{\partial t}+\frac{\partial \left(\varrho {\bar{u}}_{i}{\bar{u}}_{j}\right)}{\partial x_{j}}=-\frac{\partial \bar{p}}{\partial x_{i}}+\frac{\partial }{\partial x_{j}}\left(\mu \left(\frac{\partial {\bar{u}}_{i}}{\partial x_{j}}+\frac{\partial {\bar{u}}_{j}}{\partial x_{i}}\right)\right)-\frac{\partial \left(\varrho \bar{{u^{\prime }}_{i}{u^{\prime }}_{j}}\right)}{\partial x_{j}}$$

where *ϱ* is density, ${\bar{u}}_{i}$ is *i*-th component of mean velocity, $\bar{p}$ is mean pressure, *μ* is dynamic viscosity of the fluid, ${u’}_{i}$ is *i*-th component of fluctuation velocity, and $\bar{{u^{\prime }}_{i}{u^{\prime }}_{j}}$ is Reynolds stress tensor. The individual terms of the equation represent: $\frac{\partial \left(\varrho {\bar{u}}_{i}\right)}{\partial t}$ is the unsteady term, which corresponds to the temporal rate of change of mean momentum, $\frac{\partial \left(\varrho {\bar{u}}_{i}{\bar{u}}_{j}\right)}{\partial x_{j}}$ is the convective term—the transport of mean momentum by the mean flow, $-\frac{\partial \bar{p}}{\partial x_{i}}$ is the pressure gradient, which is defined as the force induced by the mean pressure, $\frac{\partial }{\partial x_{j}}\left(\mu \left(\frac{\partial {\bar{u}}_{i}}{\partial x_{j}}+\frac{\partial {\bar{u}}_{j}}{\partial x_{i}}\right)\right)$ is the viscous term, which accounts for forces arising from fluid viscosity, and $-\frac{\partial \left(\varrho \bar{{u’}_{i}{u’}_{j}}\right)}{\partial x_{j}}$ is the Reynolds stress, which signifies the supplemental momentum transport induced by turbulent fluctuations.

The primary challenge associated with the RANS equations lies in the Reynolds stress tensor. This term introduces more unknowns than there are available equations, rendering the system open. To achieve closure of this system, turbulence models are employed. In this specific instance, a model predicated on Boussinesq’s hypothesis was utilized, positing a linear relationship between the Reynolds stress tensor and the mean rate-of-strain tensor. A two-equation SST k-ω model was selected, where the first equation governs the turbulent kinetic energy (*k*), encompassing its generation, transport, and dissipation, and the second equation addresses the specific dissipation rate (*ω*).

The SST k-omega model was employed for modeling flows characterized by shock waves and significant wall friction, owing to its proficiency in accurately resolving the boundary layer. Nevertheless, to achieve a robust solution for thermal energy and to accurately predict temperature increments within these regions, it is imperative to activate and appropriately account for the Viscous Heating term. Notably, substantial energy dissipation occurs due to compression and friction within the shock wave. Therefore, the activation of Viscous Heating is essential for the precise prediction of the temperature rise downstream of the shock wave, which is critical for maintaining an accurate thermodynamic balance of the flow.

A crucial element in the system configuration is the selection of an appropriate discretization solution. From the schemes available within the Ansys Fluent system, the Second-order [28] and Third-order MUSCL [29] schemes were chosen for implementation.

In this instance, comparative analyses were conducted, demonstrating that the Second-order scheme performed adequately up to approximately 6000 Pa. At lower pressures, the Third-order scheme exhibited a tendency towards more rapid convergence. In all cases, both schemes yielded consistent results.

Given the nature of supersonic flow, viscous dissipation effects were incorporated into the energy equation by activating the corresponding terms. Viscous Heating becomes significant as the Brinkman number (*Br*) approaches unity [27].

(17) $$B_{r}=\frac{\eta v_{e}^{2}}{k\Delta T}$$

where η [Pa·s] is dynamic viscosity, *k* [W·m^−1^·K^−1^] is thermal conductivity, ΔT [K] is the temperature difference in the system.

**Figure 6:**
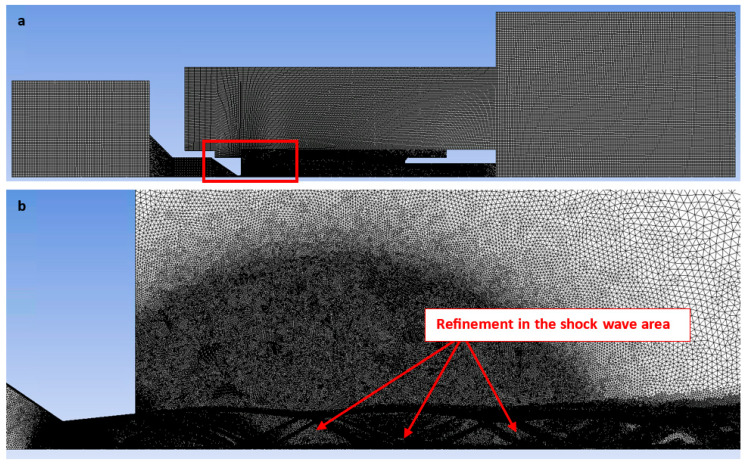
Structured mesh for CFD analyses (**a**) with zoomed refined area (**b**).

A structured mesh combining 2D structured rectangular elements with unstructured triangular elements was implemented. This approach mitigated numerical artifacts associated with skewed interfaces and optimized the cell count in predominantly rectangular regions (Figure 6). Triangular elements were utilized in regions where structured meshing was infeasible, particularly within the narrow nozzle aperture and anticipated supersonic jet zones with significant pressure and density gradients. A sufficiently fine boundary layer was modeled within this aperture and nozzle [30,31,32]. Figure 6a illustrates the expanded rectangular domain with an enlarged inset highlighting (red frame) the refined mesh region (Figure 6b). Furthermore, manual adaptive refinement based on pressure gradient criteria was applied during the computation process using the Field Variable method. The refinement was targeted at regions exhibiting oblique and normal shock waves. Instead of uniformly refining the entire mesh, which would result in an exorbitant increase in cell count and computational expense, Adaptive Mesh Refinement (AMR) dynamically adds cells only where they are most critically required. This strategy optimizes the balance between solution accuracy and computational efficiency. This refinement process was continued until further refinement yielded no discernible impact on the solution.

Mesh adaptation was controlled by maximum values, according to the solver used, of either the pressure gradient or the density gradient, utilizing a cell-based derivative approach and a maximum refinement level of 4. The setting of “maximum refinement level of 4” dictates the maximum number of times an initial cell can be recursively subdivided. Each instance of refinement corresponds to a further subdivision of a cell into smaller constituent cells. This effectively captured the pressure gradients within the supersonic nozzle flow. The resulting mesh exhibits higher density in areas where numerical precision is paramount (e.g., at shock waves or flow separation points) and coarser density in regions of relatively constant pressure. This approach ensures an accurate solution with efficient utilization of computational resources.

The arithmetic average roughness, denoted *Ra*, is most commonly used to determine surface roughness. It is a characteristic of the material surface that describes the surface irregularities at a microscopic level. *Ra* values are standardized as follows: 0.4; 0.8; 1.6; 3.2; 6.3…

In the Ansys Fluent system, the direct application of *Ra* values is not feasible; instead, the concept of sand-grain roughness is employed. Consequently, a conversion from *Ra* values to sand-grain roughness values is necessary. Sand-grain roughness is a conceptual parameter representing surface roughness based on its impact on fluid flow, typically derived from empirical correlations. It reflects an equivalent roughness height that would induce a similar resistance to flow as the actual surface. While sand-grain roughness primarily influences boundary layer development and friction factor in turbulent flows, geometric roughness can induce laminar-turbulent transition. Sand-grain roughness is not a direct physical measurement. It is inferred from experimental data by comparing the actual flow resistance of a surface to that of an idealized sand-grain rough surface. The estimation of sand-grain roughness (*ε*) requires the use of empirical or semi-empirical methods based on measured data and is performed outside the Ansys Fluent system (Equation (16)). These equivalent sand-grain roughness values are not derived from direct measurements of surface roughness obtained through standard surface characterization equipment or its associated definitions. In instances where direct surface roughness data for a pipe surface is unavailable, particularly with novel materials or fabrication methods, direct measurement using profilometers is the conventional approach. Consequently, establishing a correlation between directly measured surface roughness and the sand-grain roughness relevant to friction factor calculations would be highly beneficial for academic purposes. Further detailed information can be found in [33].

(18) ε=5863Ra

### 2.3. Experimental Measurement Settings

**Figure 7:**
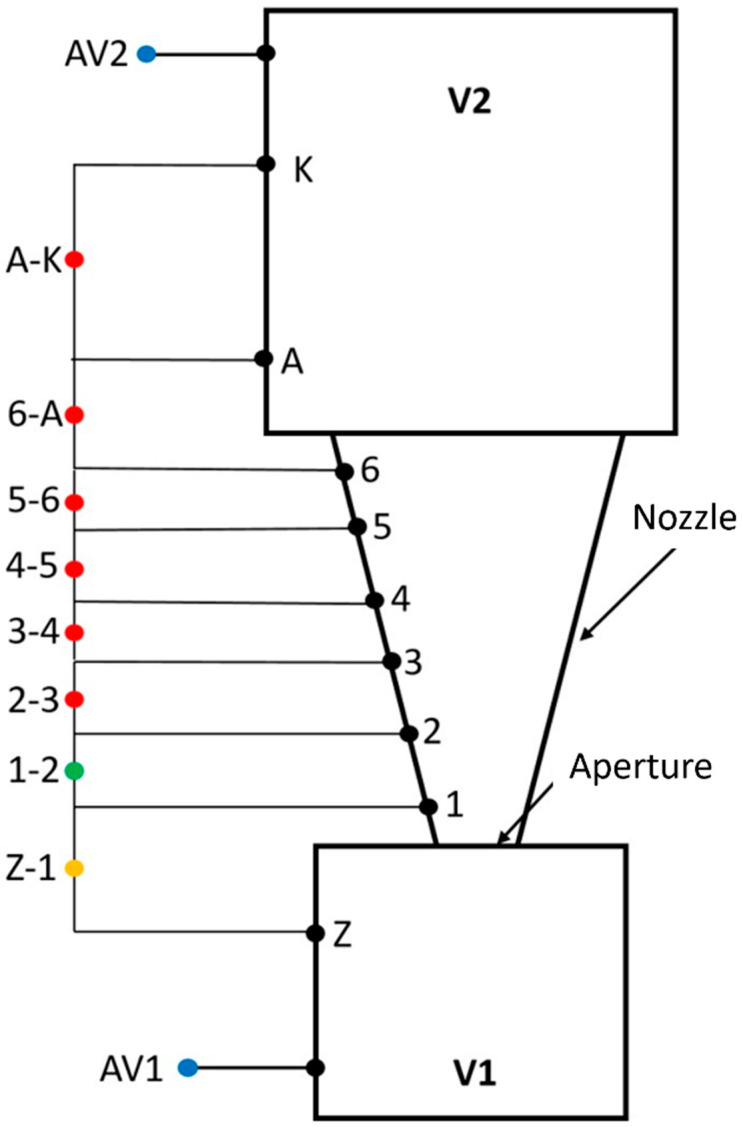
Schematic arrangement of the pressure sensors.

Experimental pressure measurements were conducted within an experimental chamber, following the schematic depicted in Figure 7. These pressure sensors were meticulously selected to accommodate the substantial pressure gradients, flow velocity, experimental chamber dimensions, and other relevant factors. Ultimately, absolute pressure sensors from Pfeiffer Vacuum were chosen, specifically the Pfeiffer CMR 361 and Pfeiffer CMR 362 models [34]. For pressure measurements along the nozzle wall, differential pressure sensors from BD Sensors, s.r.o., were employed, namely the DPS 300 series with varying ranges [35].

Figure 7 illustrates a schematic representation of the experimental chamber, depicting the schematic arrangement of the pressure sensors (this is a schematic only and not to scale). The description of the pressure sensors and measured points is given in Table 1.

## 3. Results

### 3.1. Experimental Measurement of Static Pressure Within the Experimental Chamber

The initial experimental measurement of static pressures on the nozzle wall was conducted on the above-mentioned experimental chamber. A schematic representation of the pressure measurement locations within the experimental chamber is depicted in Figure 6. It is important to note that the experimental measurements on this nozzle were performed under underexpanded nozzle conditions for a pressure ratio of V1:V2 = 10:1. This was motivated by the objective of generating oblique shock waves within the nozzle, which are analyzed in this experiment to investigate the influence of the modified ratio of inertial and viscous forces.

The results of the experimental measurements are presented in Table 2. The experimental measurement variants that will be subsequently compared with CFD analyses are indicated in the column labeled “Abs. pressure in V1”. This designation of variants will be consistently used hereafter. Nevertheless, for all variants, an effort was made during the experiment to maintain an approximately consistent pressure ratio between chambers V1 and V2, specifically a pressure ratio of 10:1.

**Figure 8:**
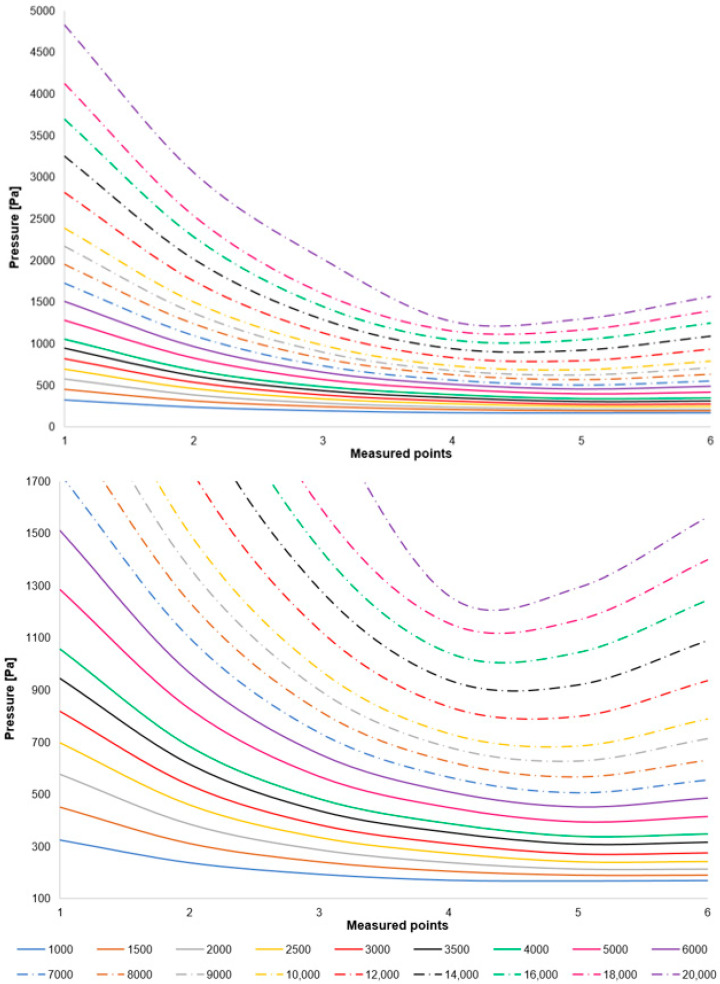
Comparison of the pressure layout of individual variants across the entire range (**top**) and with a modified scale (**bottom**).

A comparison of the layout of these variants is depicted in the graph in Figure 8 top. For enhanced illustration of the phenomenon occurring around measurement point 4, the scale in Figure 8 bottom has been modified. Initial analyses of the results demonstrate that the oblique shock wave impinging upon the boundary layer within the nozzle induces a pressure increase (to be discussed further). It is evident that the altered ratio of inertial to viscous forces under low pressure conditions influences the characteristics of this shock wave. This manifests as a reduced impact of the shock wave on the boundary layer within the nozzle, to the extent that for variants below 3000 Pa, this increase does not occur, or alternatively, this discontinuity shifts away from the aperture towards the nozzle throat and becomes negligible.

Eight repeated experimental measurements of pressure differentials were conducted for the aforementioned pressure ratios, and the averaged values were utilized for further analysis. To evaluate the measurement error, the Standard Error of the Mean (*SEM*) methodology was employed. The *SEM* represents the standard deviation of the sampling distribution of the sample mean, derived from data obtained as a random sample from the population. It is a quantity that indicates the extent to which the obtained sample mean is likely to deviate from the true population mean.

The evaluation was performed based on Equation (19):

(19) $$SEM=\frac{\sigma }{\sqrt{n}}$$

where *σ* [Pa] is the standard deviation of the sample, and *n* is the quantity of data points from which the mean was evaluated.

As presented in Table 3, which provides an example for the 2000 Pa variant, the mean error of the average did not exceed 1.5 Pa. The labels in Table 3 correspond to those in Figure 7.

Subsequently, a comparison of the selected experimental result variants with the CFD analyses was conducted. This comparison, along with the measurement error, is presented in Table 4.

**Figure 9:**
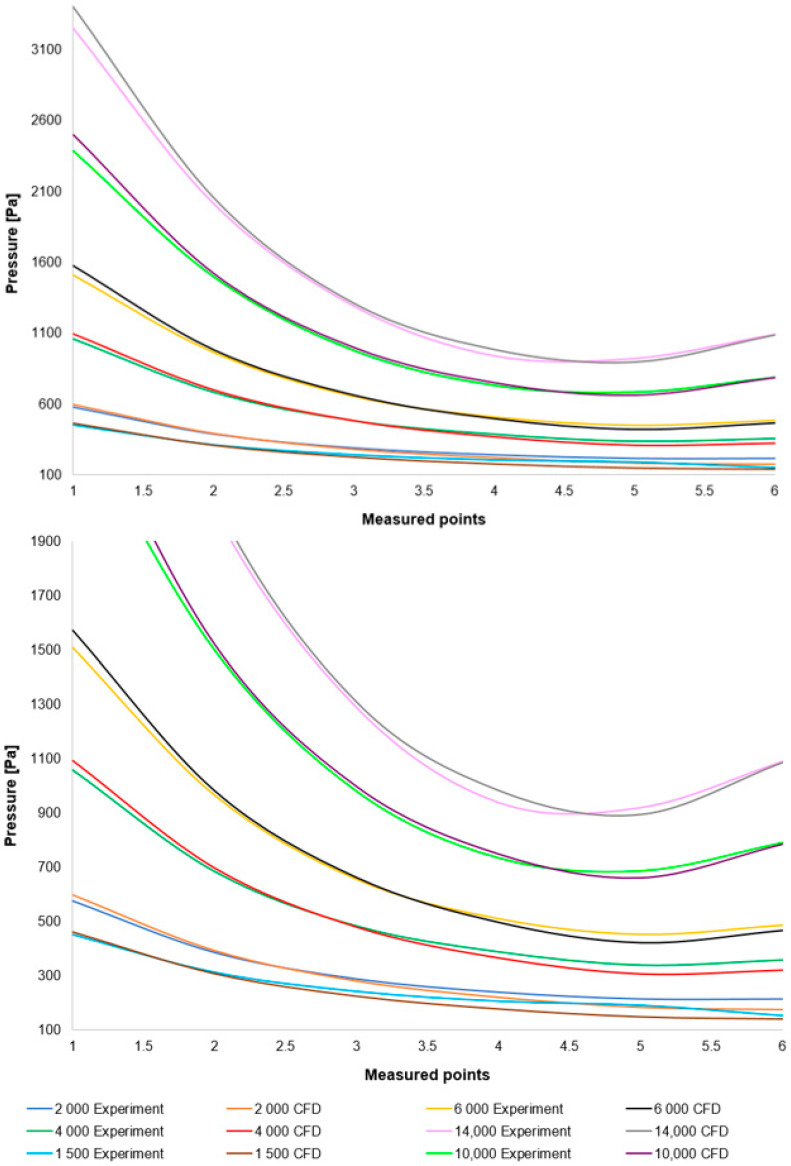
Pressure layout for individual variants, comparing CFD simulations with experimental measurements across the entire range (**top**) and with a modified scale (**bottom**).

This comparison is depicted in Figure 9, top, alongside a graph with a modified scale for enhanced clarity (Figure 9, bottom). This comparison reveals a relatively small error, which is considered acceptable given that a deviation of up to 20% signifies a successful measurement. This demonstrates that the fine-tuned variant within the Ansys Fluent system, which was ultimately employed, is appropriate for the given type of physical conditions in the differentially pumped chamber calculation.

Naturally, further investigation will be conducted into the reason for the elevated error observed in the region downstream of the oblique shock wave in low-pressure variants. This discrepancy stems from the fact that the flow, as recorded experimentally, exhibits lower velocities than those predicted by the results obtained from the Ansys Fluent system. This phenomenon will constitute a part of our subsequent research, where it is likely that the ratio of inertial to viscous forces plays a significant role, even within the realm of continuum mechanics.

The results presented in this manuscript will furthermore serve as a foundation for subsequent experiments. These will include pressure measurements on the nozzle walls and along the flow axis, utilizing nozzles with precisely defined roughness. The selection of these experiments will be based on the outcomes achieved in this manuscript.

Following this, two specific variants, incorporating the defined nozzle wall roughness as detailed in the Methodology chapter, were subjected to comparative analysis. The results demonstrated that the inclusion of nozzle wall roughness led to an even closer agreement between the CFD analysis and experimental findings. A more comprehensive discussion of these observations, including a detailed analysis of these CFD variants, will be presented later in this paper.

### 3.2. The Influence of Surface Roughness in the 10,000 Pa Variant

Analyses were conducted with nozzle wall roughness set at fundamental roughness scale values: No roughness, Ra = 1.6, Ra = 3.2, and Ra = 6.3. Subsequently, these analyses were compared with experimental pressure measurements obtained from the surface of a nozzle manufactured with a production tolerance of Ra = 1.6.

The values presented in Table 5 were obtained through CFD analyses, wherein the nozzle wall roughness was defined according to the methodology detailed above. Concurrently, the table also includes the relative measurement error and the average relative measurement error for the entire roughness variant. The results indicate that the Ra = 6.3 variant exhibited the largest error in relation to the experimentally acquired value. The second largest error was observed in the No roughness variant. Thus, roughness has a discernible influence on the accuracy of CFD simulations, although the error appears negligible. The experimental nozzle was manufactured with a roughness of Ra = 1.6, which resulted in a CFD analysis error of less than 1%. Surprisingly, variants with higher roughness (Ra = 3.2) demonstrated even smaller errors, with the Ra = 3.2 variant being the most accurate. This effect may be attributed to the possibility that the manufactured nozzle’s roughness was closer to the Ra = 3.2 value within manufacturing tolerances. However, it appears more likely that, within the spherical roughness modeling methodology, the Ra = 3.2 roughness setting corresponds more accurately to the actual manufactured roughness of Ra = 1.6, which possesses sharper features. The premise that the actual roughness may be higher will be rigorously investigated in a subsequent study utilizing newly manufactured nozzles with precisely defined roughness characteristics. Nevertheless, it may be demonstrated that a spherical roughness, when applied to the problem of boundary layer detachment, exerts a less significant effect than the actual roughness value Ra.

This variant demonstrated that higher roughness values exhibited agreement with experimental measurements. The data thus obtained from experimental measurements contributed to the refinement and verification of the Ansys Fluent system. Subsequently, an analysis of the flow characteristics will be presented, utilizing the results acquired from the Ansys Fluent system.

**Figure 10:**
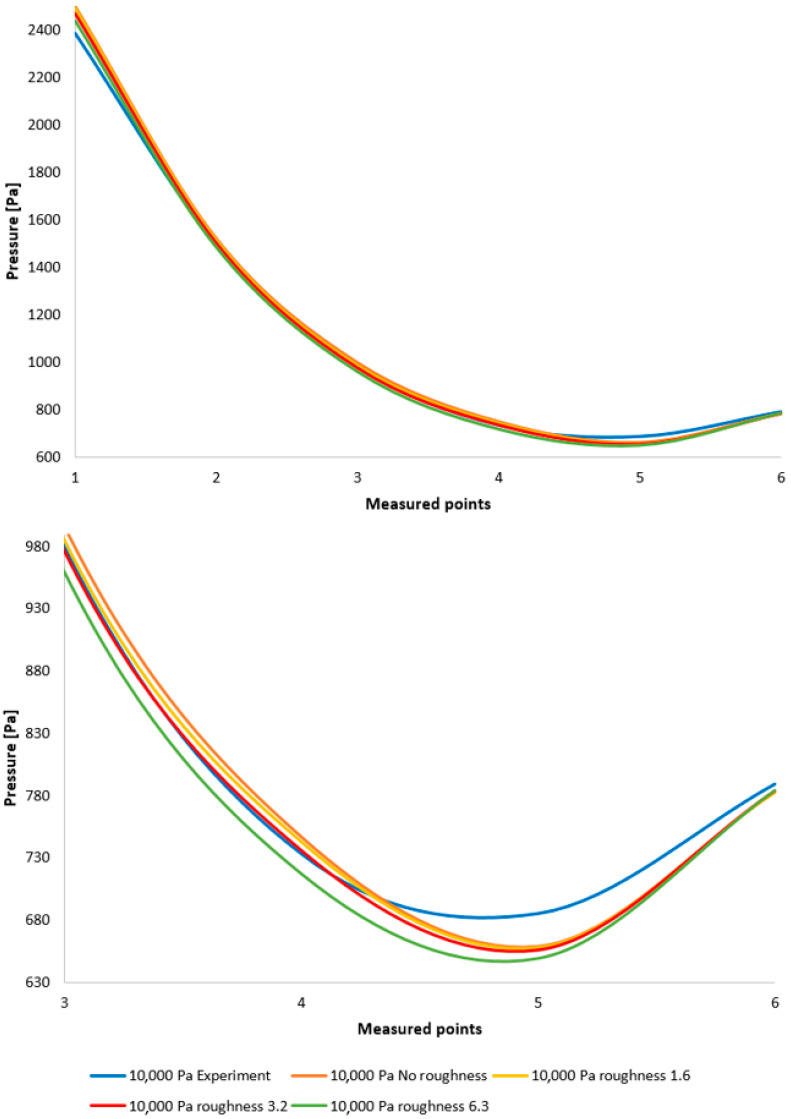
Comparison of pressure layouts for the 10,000 Pa variant obtained through experimental measurement with values derived from the Ansys Fluent system, considering the specified nozzle wall roughness at individual measurement points across the entire range (**top**) and with a modified scale (**bottom**).

A comparison between the experimental results and those obtained from CFD analyses demonstrates that the data derived from the CFD analysis exhibit a high degree of accuracy when compared to the experimental measurements. The correlation between the experimental results and the CFD simulation outcomes is illustrated in Figure 10.

The subsequent analysis investigated the flow field within the CFD simulations. This contemporary approach, effectively integrating experimental measurements with CFD simulations, provides significant added value to the research. In this instance, experimental data acquisition describes the distribution of variables at selected points and serves to verify the CFD simulations. Concurrently, the CFD simulations offer a detailed description of the entire domain.

**Figure 11:**
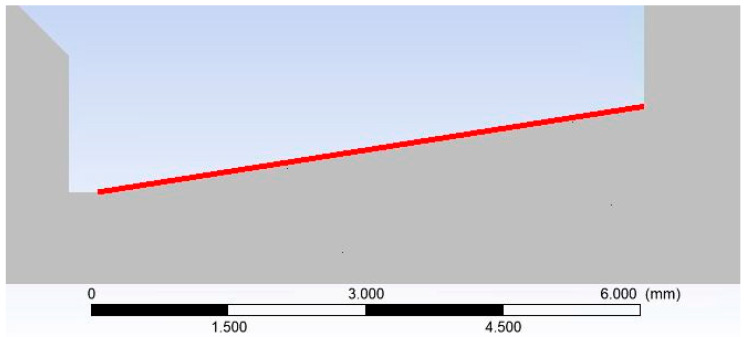
The defined path for the evaluation of physical quantities (nozzle wall).

Initially, the pressure and temperature distribution along the nozzle wall was analyzed, as the experimental pressure measurements are focused on this region at this stage of the research. Temperature measurements using sensors are planned for the near future. Figure 11 illustrates the specific path along which these quantities were extracted (referred to as “nozzle wall” in the graphs).

**Figure 12:**
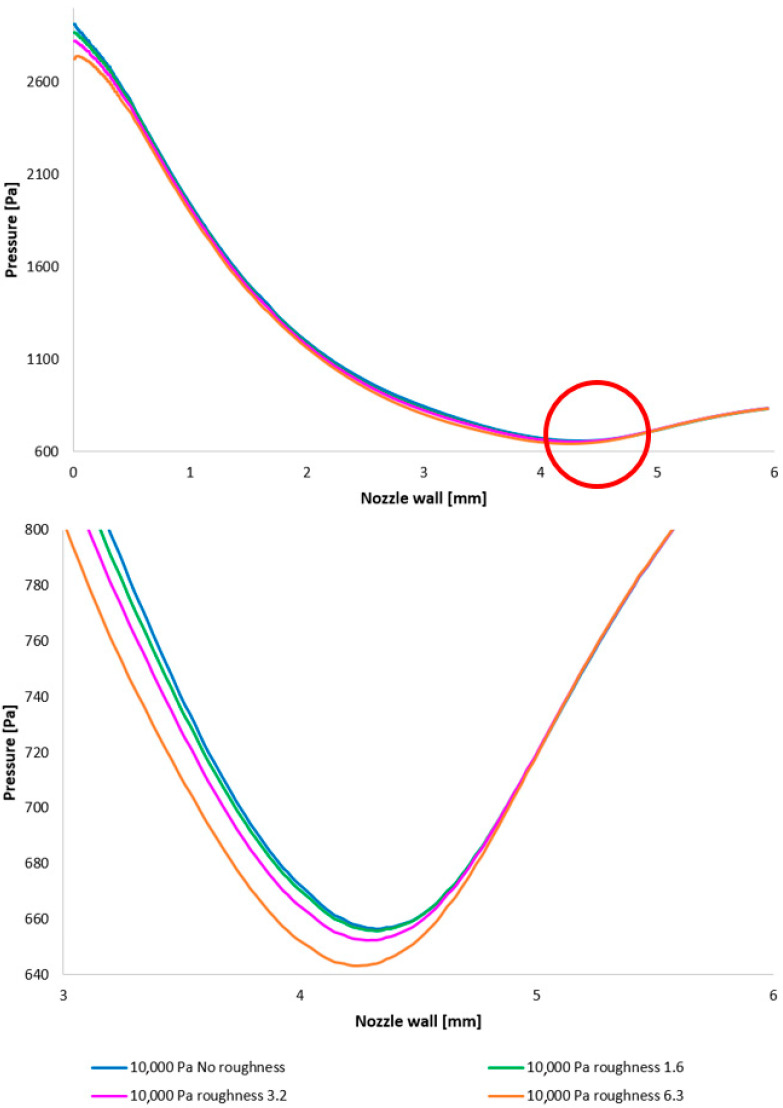
A comparison of pressure layouts for the 10,000 Pa variant with the nozzle wall roughness set across the entire range (**top**) and with a modified scale (**bottom**).

Figure 12 illustrates the static pressure distribution along the nozzle wall. A discontinuity resulting from an oblique shock wave is evident at approximately the 4.3 mm position. The region of this discontinuity, encircled in red, is presented in magnified form in Figure 12, bottom, using a modified scale to accentuate the differences between the configurations. Figure 12, bottom, employing a modified scale, clearly demonstrates that the pressure loss increases with increasing surface roughness. The difference between the No roughness variant and the Ra = 6.3 variant reaches up to 30 Pa. Furthermore, the Ra = 3.2 and Ra = 6.3 variants exhibit the smallest deviations when compared to the experimental measurements. This constitutes a significant finding for future research in the field of ESEM development.

**Figure 13:**
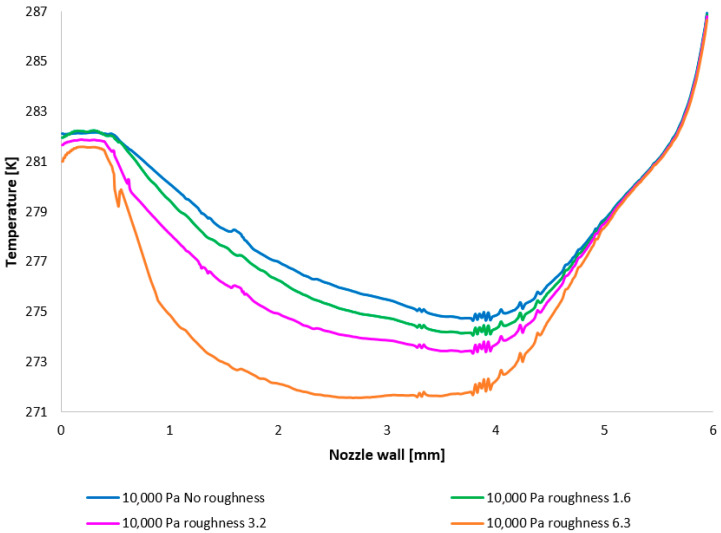
A comparison of temperature layouts for the 10,000 Pa variant with the nozzle wall roughness set across the entire range (**top**) and with a modified scale (**bottom**).

The temperature layout along the nozzle wall (Figure 13) demonstrates the anticipated correlation with the static pressure layout, thus providing a suitable basis for validation.

It is pertinent to note that on the x-axis of Figure 13, an unexpected phenomenon occurred whereby the retention of a highly granular division resulted in a jagged graphical representation. This issue was resolved through a refinement of the discretization and a subsequent revision of the results. This demonstrated that the observed irregularity was not attributable to an error stemming from a solution dependency on an inappropriately generated discretization mesh.

In this particular case, the analysis pertains to a region where the magnitude of inertial forces significantly diminishes due to low pressure, whereas the viscous forces remain independent of pressure magnitude up to 133 Pa. Consequently, in the scenario under investigation, viscous forces exhibit significant dominance.

When inertial forces are negligible relative to viscous forces, the flow tends to exhibit laminar characteristics. In such flow regimes, the boundary layer is generally less stable and highly susceptible to separation in the presence of an adverse pressure gradient. This phenomenon is particularly pertinent in cases where a shock wave impinges upon the boundary layer within the divergent section of the nozzle. A separation of the laminar boundary layer would result in substantial pressure losses and a reduction in the nozzle’s effective cross-sectional area.

Under these conditions, the increased wall roughness in this specific case influences the boundary layer by inducing a premature transition from laminar to turbulent flow. The resulting turbulent boundary layer exhibits significantly greater resistance to separation compared to its laminar counterpart. This enhanced resilience is attributed to its higher kinetic energy near the wall, which enables it to more effectively overcome pressure increases and maintain attachment to the surface.

If roughness prevents boundary layer separation, the flow remains more effectively attached along the entire length of the nozzle. This maintains a larger effective cross-section for the flow and minimizes losses caused by recirculation regions that arise from separation. Consequently, the flow can achieve a higher velocity.

This case is highly specific due to the significantly different ratio of inertial to viscous forces, where increased roughness leads to improved conditions for boundary layer stability.

**Figure 14:**
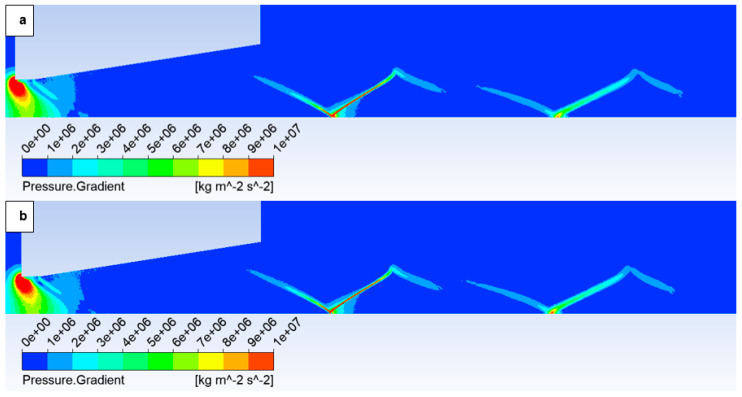
Pressure gradient distribution in the nozzle for variant of 10,000 Pa: No roughness (**a**) and Ra = 6.3 (**b**).

**Figure 15:**
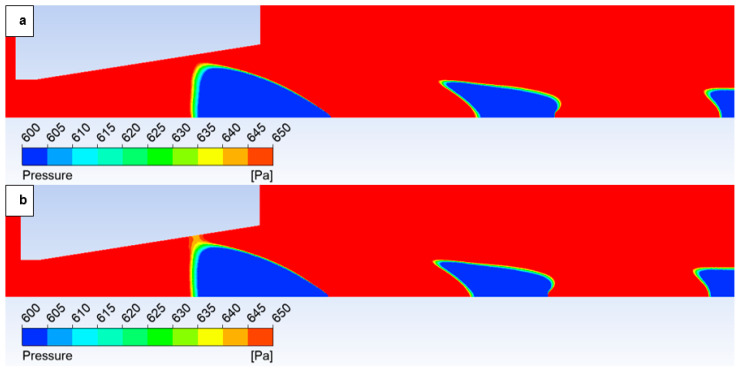
Pressure distribution in the nozzle, encompassing the boundary layer-affected region for the variant of 10,000 Pa: No roughness (**a**) and Ra = 6.3 (**b**).

The oblique shock wave referenced above is illustrated in Figure 14, which depicts the pressure gradient distribution. The following figures show both variants of the opposite spectrum, i.e., the No roughness variant (Figure 14a) and the Ra = 6.3 variant (Figure 14b). Figure 14 shows the character of an oblique shock wave using a pressure gradient, the influence of which decreases with decreasing pressure. This shock wave influences the boundary layer (Figure 15a). An enlarged view of this region with a modified scale is presented in Figure 15b, highlighting these pressure gradients.

However, with a suitable scale setting, it is evident in Figure 15 that for the variant with roughness Ra = 6.3 (Figure 15b), the shock wave has a greater tendency to interact with the boundary layer, which results in the above-mentioned influence on the pressure and temperature profile on the nozzle wall. This interaction influences the subsequent analysis in the flow axis.

Analysis of the 10,000 Pa variant reveals a distinct interaction between the oblique shock wave and the boundary layer. This interaction influences the evolution of state variables along the flow axis due to boundary layer separation. Larger and sharper surface irregularities exert a more significant impact on this separation. The influence of surface roughness increases with augmenting flow velocity. However, this effect is less pronounced with decreasing pressure, as will be demonstrated subsequently in the analysis of the 2000 Pa variant.

**Figure 16:**
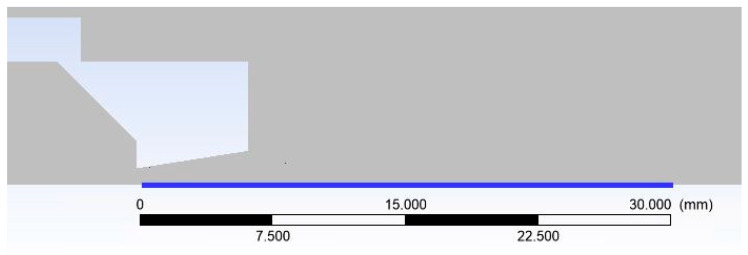
Path for quantities evaluation (Flow axis).

Subsequent analysis presents the flow profile along the nozzle axis, which may be influenced by boundary layer behavior. Figure 16 illustrates the trajectory along the flow axis, upon which selected state variables are subsequently plotted (denoted as “Flow Axis” in the graphs).

**Figure 17:**
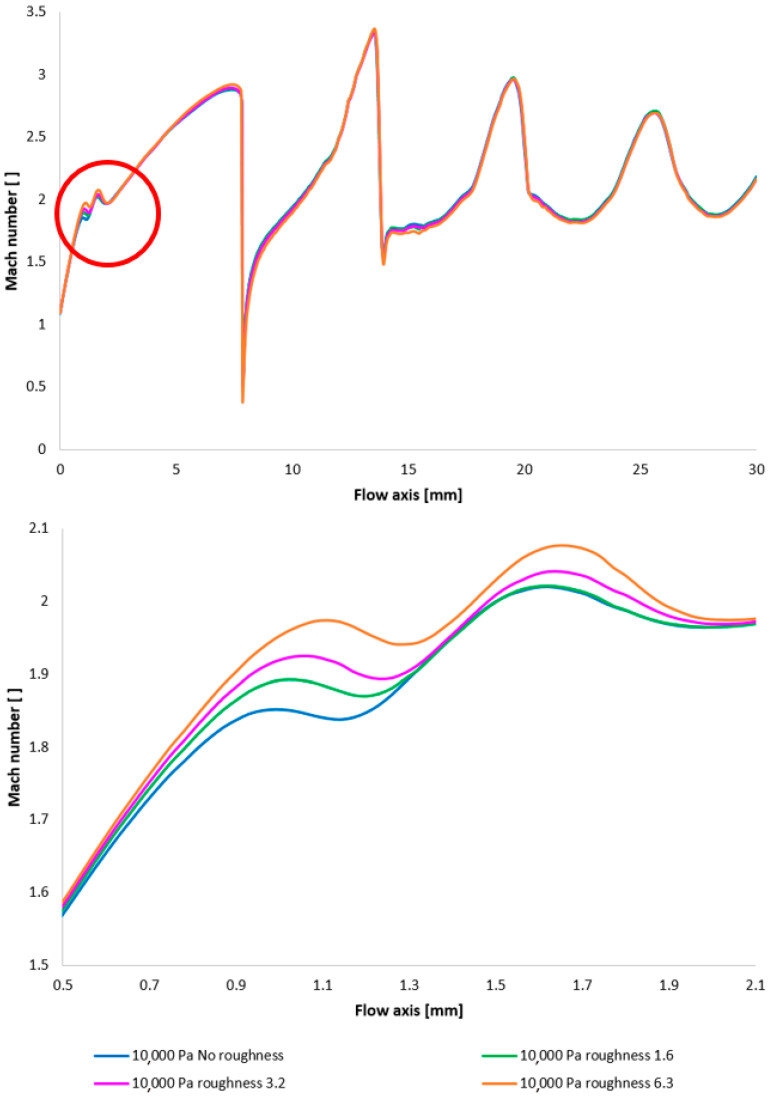
A comparison of Mach number layouts for the 10,000 Pa variant with the nozzle wall roughness set in the flow axis across the entire range (**top**) and with a modified scale (**bottom**).

Figure 17 illustrates the Mach number distribution, with a highlighted region affected by shock wave interactions with the boundary layer, as previously described. The area impacted by these shock waves is indicated by a red circle in Figure 17, top. Figure 17, bottom, provides a magnified view of this region, revealing flow alterations within the nozzle due to oblique shock waves. The results suggest that increased surface roughness leads to a higher flow velocity downstream of the aperture until the velocity decreases due to the formation of an oblique shock wave, which causes a velocity reduction but not below 1 Mach. As evidenced by the results presented in Figure 17, an increase in velocity downstream of the aperture, prior to deceleration induced by the shock wave, is observed to be 8.25% with increasing roughness.

**Figure 18:**
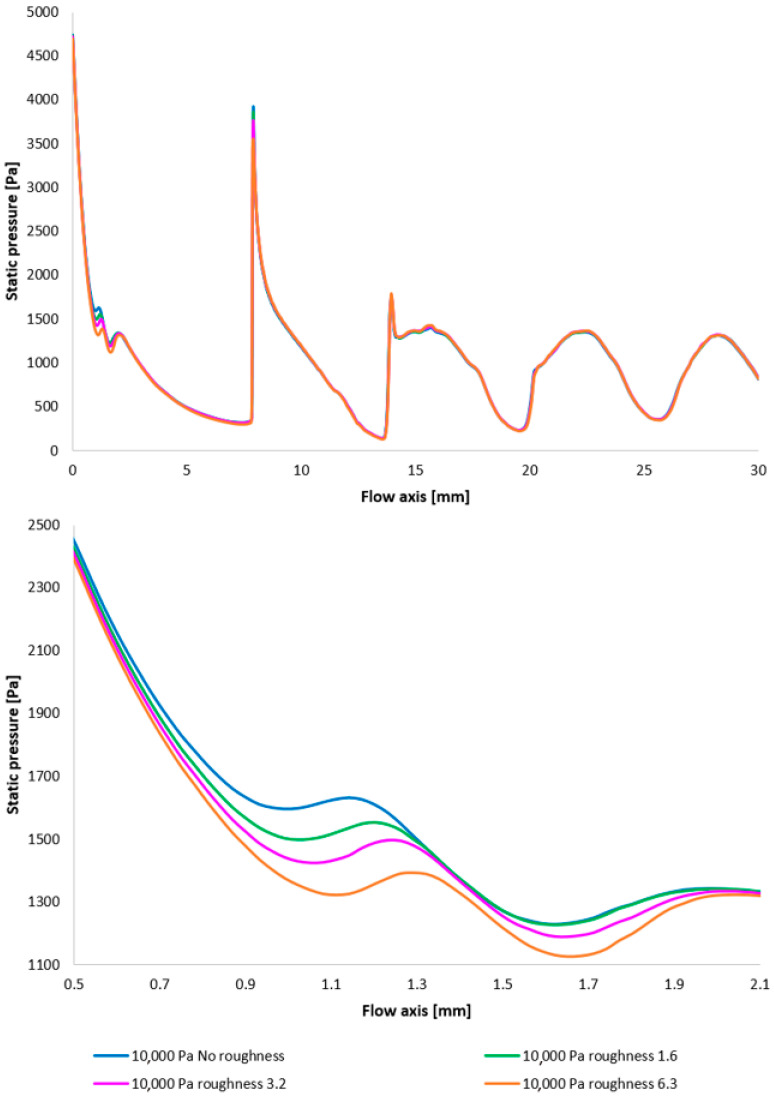
A comparison of pressure layouts for the 10,000 Pa variant with the nozzle wall roughness set in the flow axis across the entire range (**top**) and with a modified scale (**bottom**).

The Mach number distribution indicates that this phenomenon results in a more pronounced pressure drop upstream of the oblique shock wave (Figure 18 with the enlarged, red-circled region in Figure 18, bottom). This finding may have implications for the electron beam scattering upon traversing the differentially pumped chamber. This is partly due to the aim of achieving a lower average static pressure along the flow axis, but primarily because this region is in close proximity to the aperture, which is generally an area of relatively high pressure and thus exerts a greater influence on electron beam scattering.

**Figure 19:**
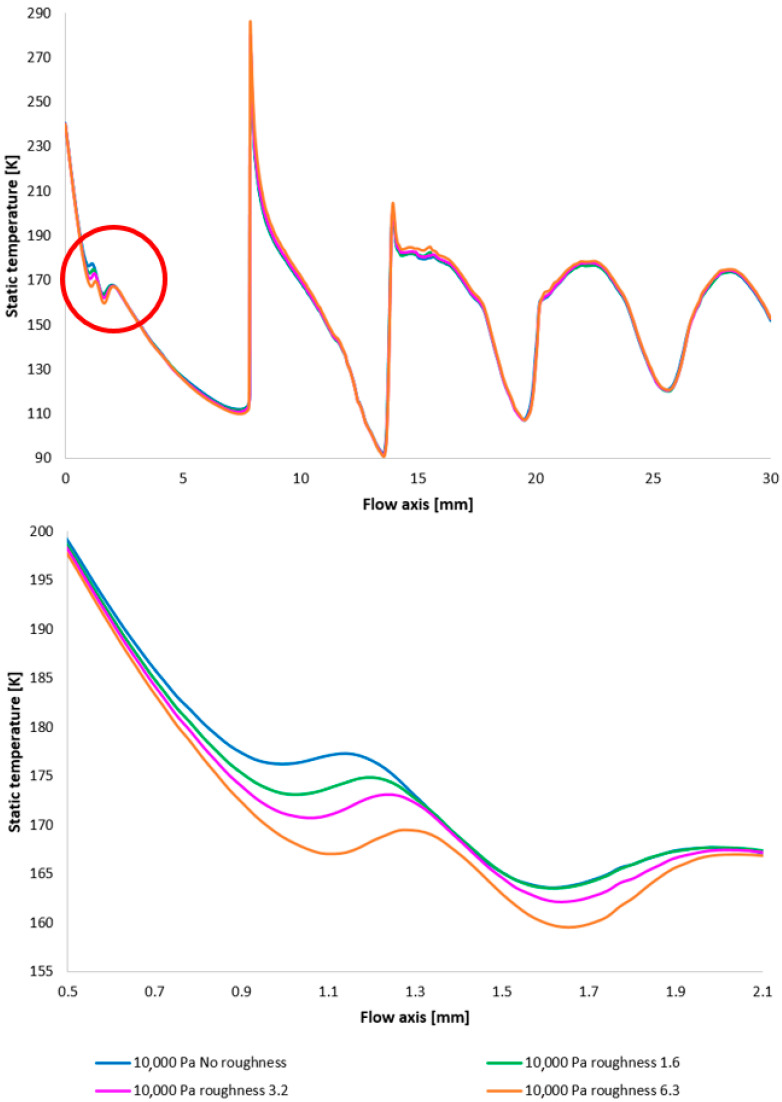
A comparison of temperature layouts for the 10,000 Pa variant with the nozzle wall roughness set in the flow axis across the entire range (**top**) and with a modified scale (**bottom**).

To facilitate a more comprehensive understanding of the flow characteristics, temperature profiles are also included, correlating with the Mach number and the static pressure distribution along the specified flow axis (Figure 19, top, with the magnified red-circled region shown in Figure 19, bottom). The temperature in regions exhibiting high Mach numbers decreases to cryogenic levels. The discrepancies observed between different variants reach up to 10 K.

In conclusion to this subsection, a cursory comparison can be made between two contrasting roughness configurations: No roughness variant and a set roughness of Ra = 6.3, as depicted in the figures illustrating the Mach number, static pressure, and temperature distributions. This is to facilitate the observation of the previously described discrepancies along the flow axis throughout the entire volume.

**Figure 20:**
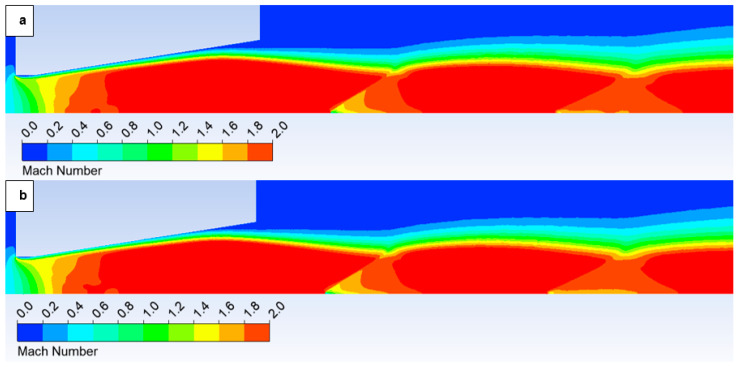
Mach number distribution in the nozzle for variant of 10,000 Pa with the roughness configuration: No roughness (**a**) and Ra = 6.3 (**b**).

Figure 20 illustrates the Mach number distribution for the No roughness variant (Figure 20a) and a variant with a surface roughness of Ra = 6.3 (Figure 20b). Within the nozzle, at the location indicated by the black circle, the previously described distinct behavior of the Mach number profile is evident. The configuration with a surface roughness of Ra = 6.3 exhibits an increase in flow velocity closer to the aperture. Boundary layer separation alters the velocity profile within the flow. In the region of separation, reverse flow develops, leading to an augmentation of velocity along the flow axis as the flow is deflected away from the surface. It is apparent that this increase primarily concerns the flow axis, through which the primary electron beam passes.

The selection of the presented range of surface roughness was motivated by the comparison of roughness influence on the flow characteristics within a small nozzle, typical of ESEM applications. The chosen scale extends from a very finely ground surface (Ra = 0.4–0.8) to a comparatively high roughness achieved through machining with a rapid feed rate (Ra = 6.3). This upper value is primarily of academic interest for the purpose of gaining insights into the flow regime, as it inherently introduces other complexities, which will be discussed subsequently.

**Figure 21:**
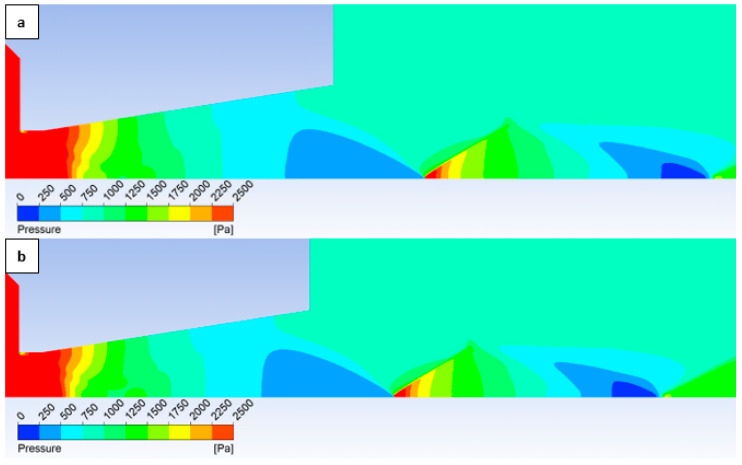
Pressure distribution in the nozzle for variant of 10,000 Pa with the roughness configuration: No roughness (**a**) and Ra = 6.3 (**b**).

Given the aforementioned dependencies between the Mach number and static pressure, Figure 21 illustrates the static pressure distribution. Notably, in the case of Ra = 6.3 (Figure 21b), a discernible pressure decrease is evident extending towards the vicinity of the aperture. Considering that the region proximal to the aperture represents the most sensitive area for electron beam scattering due to elevated pressure levels, coupled with the fact that the primary beam has traversed the longest path through non-high vacuum conditions at this point, this observation is significant [36,37].

**Figure 22:**
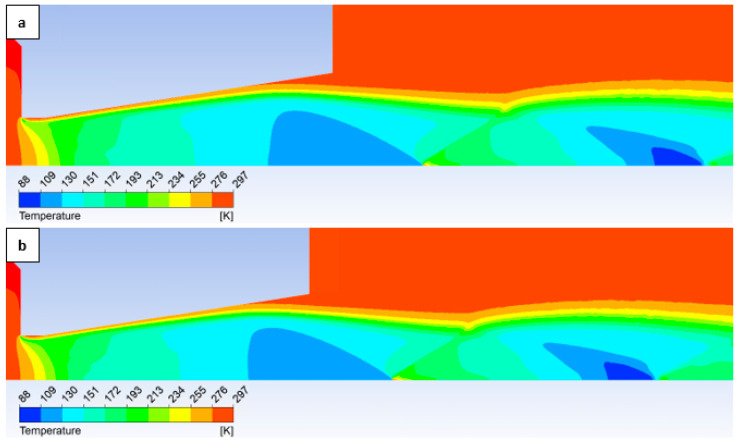
Temperature distribution in the nozzle for variant of 10,000 Pa with the roughness configuration: No roughness (**a**) and Ra = 6.3 (**b**).

To provide a complete overview, the temperature distribution is depicted in Figure 22. These findings will be significant in future work, where temperature measurements are planned utilizing temperature sensors (a custom-made Type K thermocouple with a 3 mm diameter).

### 3.3. The Influence of Surface Roughness in the 2000 Pa Variant

For the 2000 Pa variant, further analyses were conducted with selected nozzle wall roughness settings: No roughness, Ra = 1.6, Ra = 3.2, and Ra = 6.3. These are again compared with the experimental results, and this comparison is presented in Table 6.

Even in this variant, it is evident from Table 6 that the variant with a set roughness value of Ra = 3.2, followed by the variant with a set roughness value of Ra = 1.6, exhibits the smallest error. Both of these variants demonstrate an error margin of approximately 7%. In contrast to the previous configuration, further increases in the roughness value result in a larger error than the variant without roughness on the nozzle wall.

The issue concerning the higher relative error observed at certain measurement points is consistent with the description provided in Table 4.

**Figure 23:**
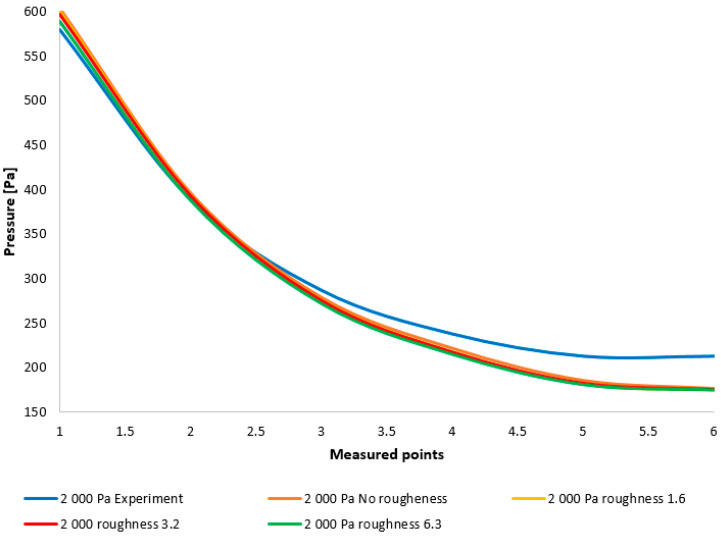
Comparison of pressure layouts for the 2000 Pa variant obtained through experimental measurement with values derived from the Ansys Fluent system, considering the specified nozzle wall roughness at individual measurement points.

Subsequently, a further analysis of the flow characteristics will be presented using results obtained from the Ansys Fluent system. Initially, Figure 23 illustrates the correlation between experimental results and CFD analysis outcomes.

**Figure 24:**
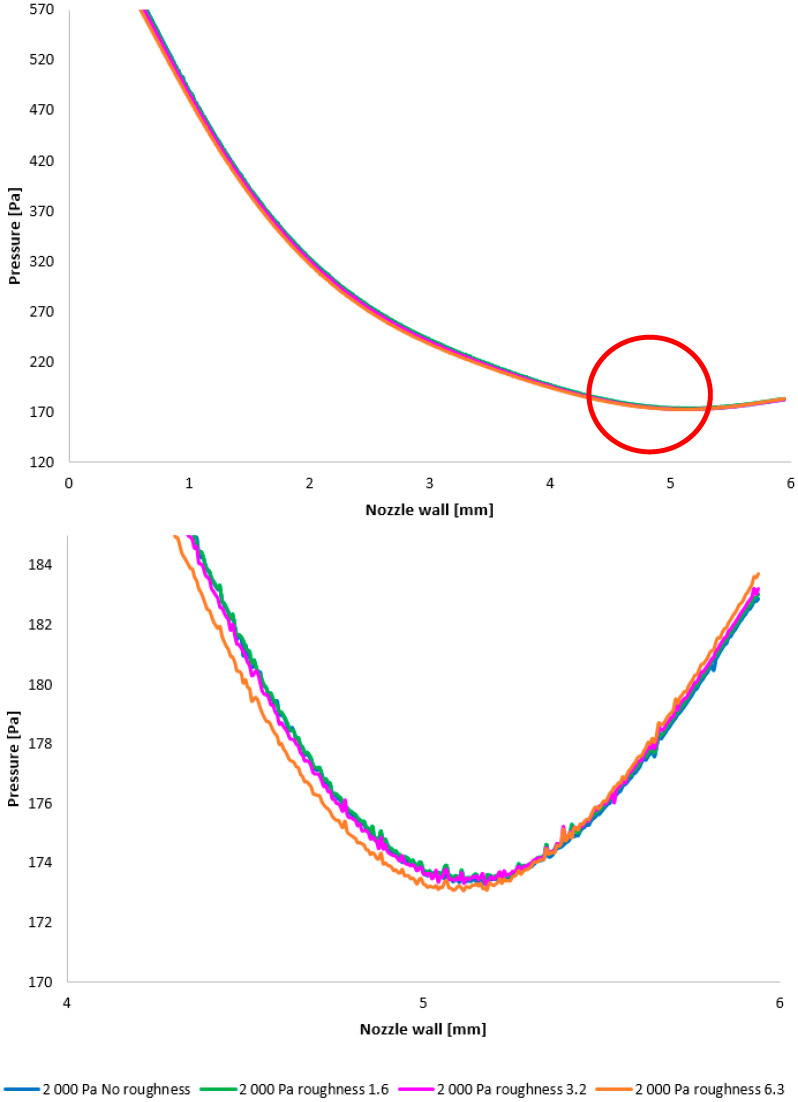
A comparison of pressure layouts for the 2000 Pa variant with the nozzle wall roughness set across the entire range (**top**) and with a modified scale (**bottom**).

In the subsequent CFD analysis, the pressure and temperature distribution on the nozzle wall were initially analyzed (Figure 11). Figure 24 illustrates the static pressure distribution along the nozzle wall. A discontinuity caused by an oblique shock wave is apparent at approximately the 4.5 mm position. The region of this discontinuity (encircled in red) is magnified in Figure 24, bottom, using an adjusted scale to emphasize the differences between the variants. Figure 24, bottom, further clearly demonstrates that, as in previous cases, the pressure loss continues to increase with increasing surface roughness; however, with the reduced pressure in this variant, this increase is less pronounced. The difference between the “No roughness” variant and the Ra = 6.3 variant is approximately 10 Pa. Again, the Ra = 3.2 and Ra = 1.6 variants exhibit the smallest deviations when compared to the experimental measurements.

**Figure 25:**
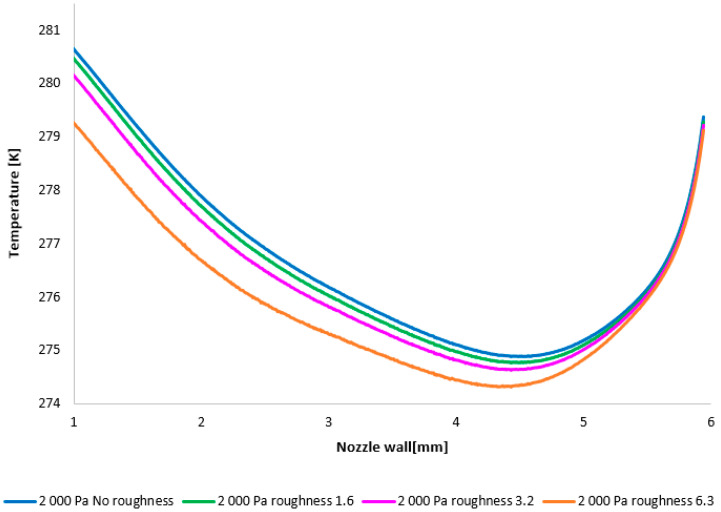
A comparison of temperature layouts for the 2000 Pa variant with the nozzle wall roughness set.

The temperature layout along the nozzle wall, as depicted in Figure 25, demonstrates the anticipated correlation with the static pressure distribution, thereby providing a basis for the aforementioned planned validation.

**Figure 26:**
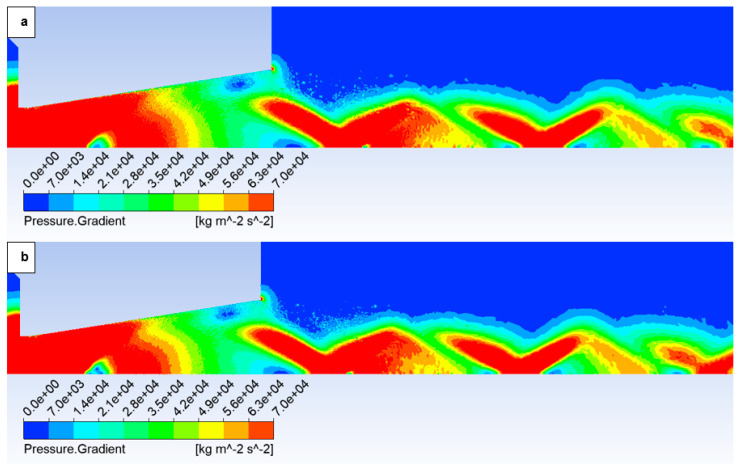
Pressure gradient distribution in the nozzle for variant of 2000 Pa with the roughness configuration: No roughness (**a**) and Ra = 6.3 (**b**).

In this variant, the shock wave weakens again, and for improved visualization, the scale in Figure 26 displaying the pressure gradient distribution had to be reduced by an order of magnitude.

**Figure 27:**
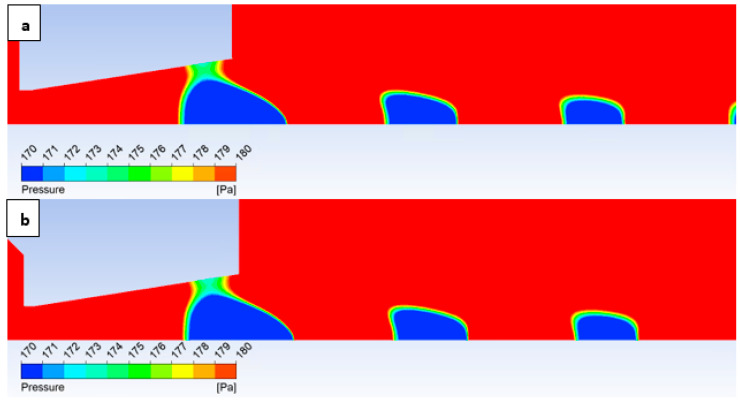
Pressure distribution in the nozzle, encompassing the boundary layer-affected region for the variant of 2000 Pa: No roughness (**a**) and Ra = 6.3 (**b**).

Consistent with the previous observation under appropriate scaling of the static pressure, Figure 27 reveals that for the variant with a roughness of Ra = 6.3 (Figure 27b), the shock wave exhibits a greater propensity to interact with the boundary layer. This interaction, as observed previously, induces alterations in the pressure and temperature profiles at the wall and consequently influences the analysis along the streamwise direction. However, the magnitude of this difference is notably diminished in this instance.

**Figure 28:**
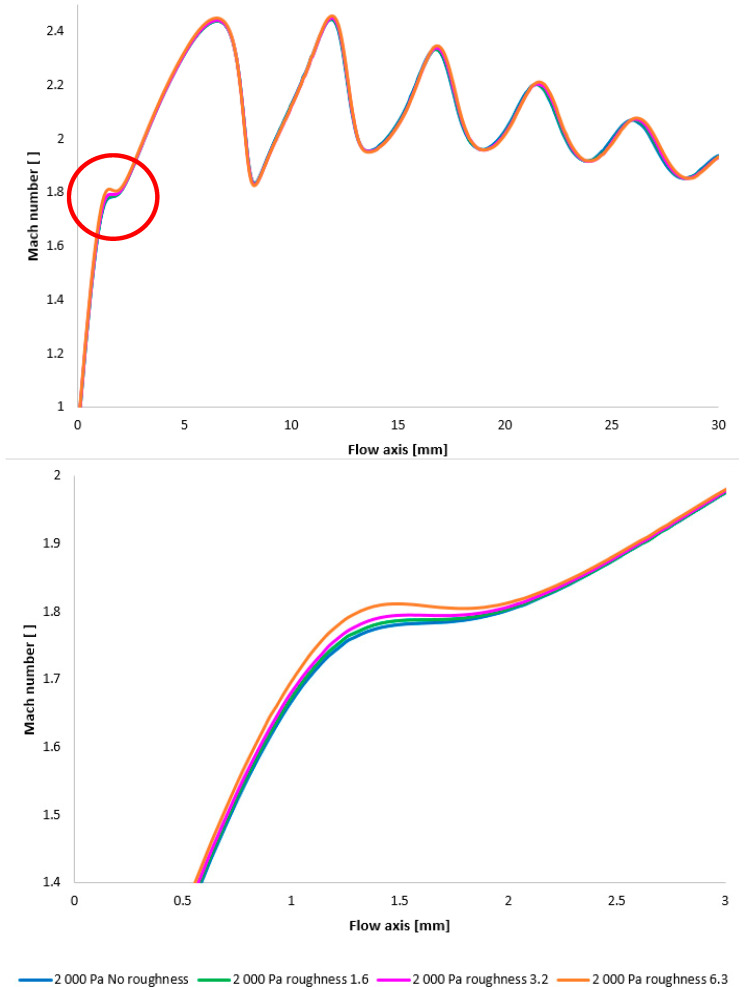
A comparison of Mach number layouts for the 2000 Pa variant with the nozzle wall roughness set in the flow axis across the entire range (**top**) and with a modified scale (**bottom**).

Figure 28 once again illustrates the Mach number distribution, with a highlighted region affected by shock wave interactions with the boundary layer, as previously described. The area impacted by these shock waves is indicated in red in Figure 28. Figure 28, bottom, provides an enlarged view of this region, revealing the flow modifications within the duct induced by oblique shock waves. Consistent with earlier findings, a higher surface roughness results in an elevated flow velocity downstream of the orifice plate until the velocity decreases due to the formation of an oblique shock wave. However, with decreasing pressure, these differences also diminish [38,39].

The results correlate with, for instance, the publication [40], where the influence of internal surface roughness on pressure drop in a small-diameter pipe was similarly determined using the Ansys Fluent system. The findings indicated a higher pressure drop in the rough pipe compared to the smooth pipe, which agrees with previously published works [41]. An increase in the wall roughness value demonstrated that further augmentation of roughness corresponds to an increase in pressure drop, albeit not proportionally. Consequently, the effect of internal surface roughness on pressure drop in small piping demonstrates its contribution to increased pressure drop. However, these analyses were conducted under standard pressure conditions. The presented paper aims to analyze this influence under low pressure, where the impact of induced drag and nozzle surface roughness on the flow may differ. Induced drag is a complex phenomenon that manifests during fluid flow around bodies. In the case of a nozzle, it refers to the resistance generated due to changes in the fluid flow direction. This change in direction induces vortices and turbulence, which dissipate a portion of the fluid’s energy, thereby increasing the overall system resistance. The surface roughness of the nozzle further complicates this phenomenon. Irregularities on the nozzle surface cause the fluid flow to become even more turbulent. Fluid particles are deflected by these irregularities, leading to the formation of additional vortices and energy losses.

Surface roughness has the potential to induce a modification in the fluid velocity profile across the nozzle’s cross-section. This alteration can consequently impact downstream flow parameters, such as pressure loss, and significantly within the area of investigation along the flow axis, through which the primary electron beam traverses in practical applications.

**Figure 29:**
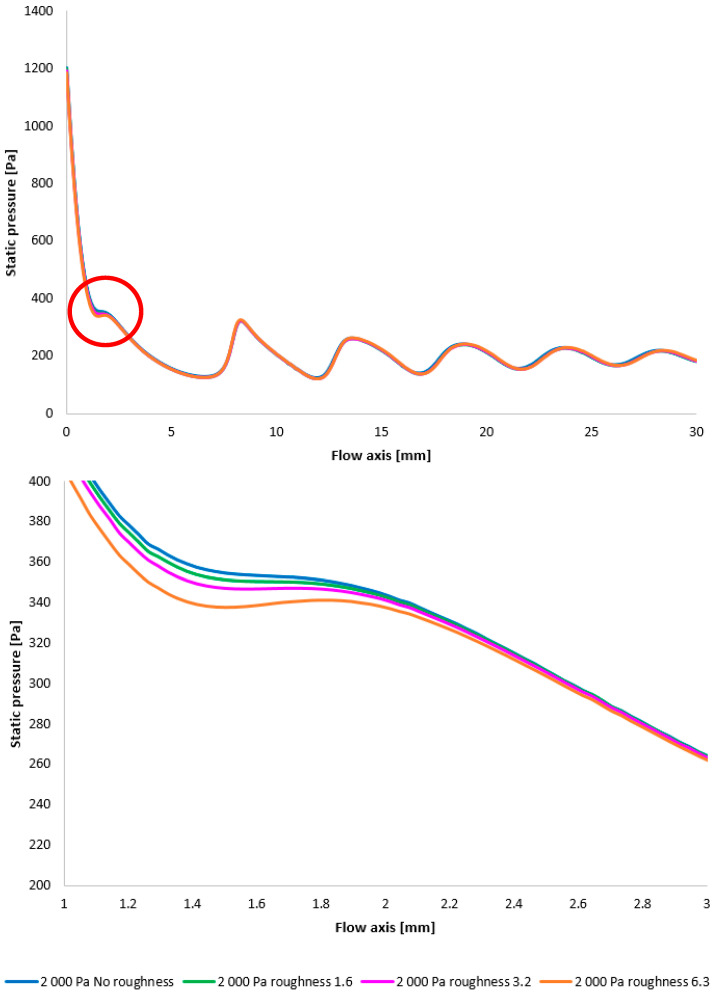
A comparison of static pressure layouts for the 2000 Pa variant with the nozzle wall roughness set in the flow axis across the entire range (**top**) and with a modified scale (**bottom**).

Again, as evident in Figure 29, the variants exhibiting a higher rate of velocity increase conversely demonstrate a more significant pressure drop, approximating over 100 Pa in the vicinity of the diaphragm. However, this difference is again less pronounced compared to the variants with higher pressure.

**Figure 30:**
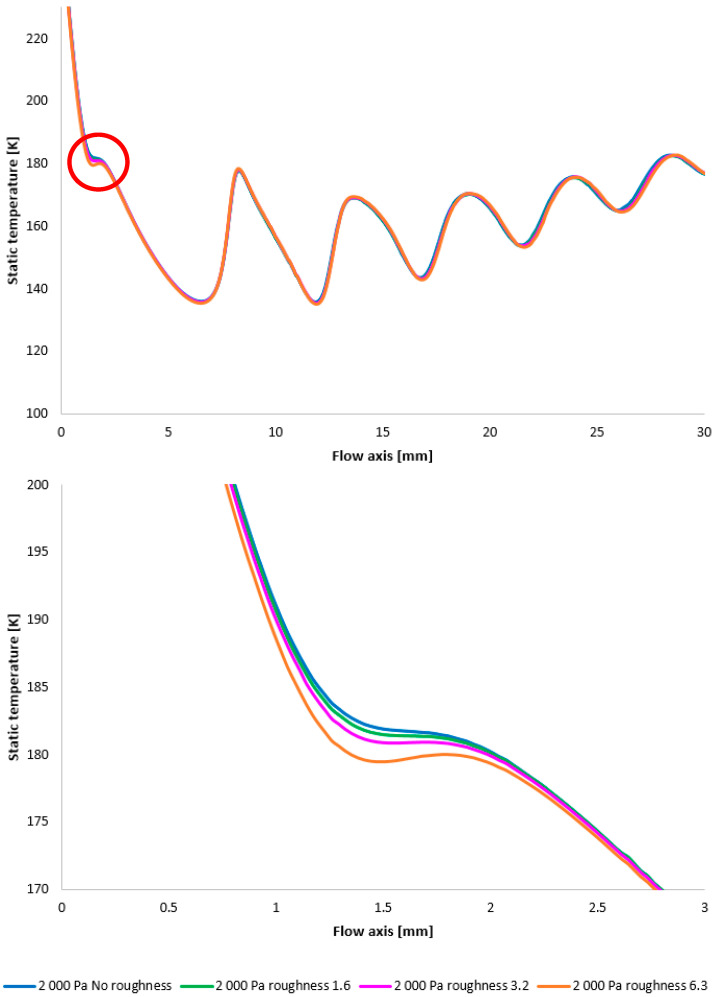
A comparison of static temperature layouts for the 2000 Pa variant with the nozzle wall roughness set in the flow axis across the entire range (**top**) and with a modified scale (**bottom**).

Furthermore, temperature profiles correlating with the Mach number and the static pressure distribution along the specified flow axis (Figure 30) are also included as a basis for subsequent planned experiments. The temperature in regions exhibiting high Mach numbers decreases to cryogenic levels.

In conclusion of the analysis of the given variant, a cursory comparison can also be made between two contrasting scenarios: the “No roughness” variant and the variant with a set roughness of Ra = 6.3, as depicted in the Mach number, static pressure, and temperature distribution plots.

**Figure 31:**
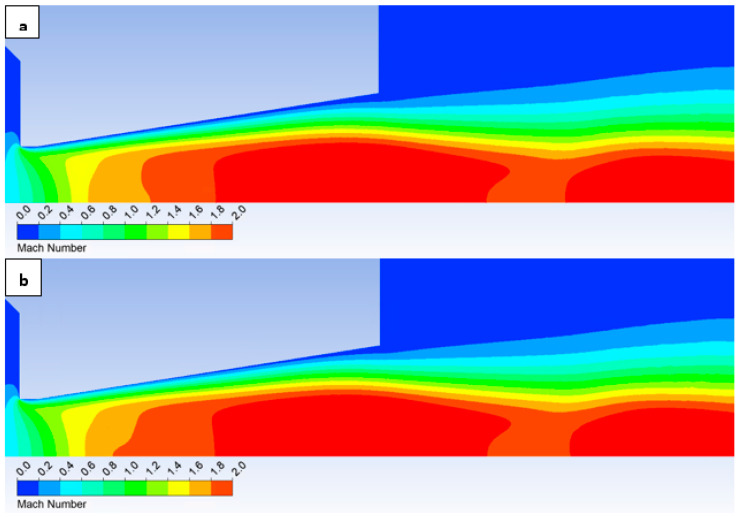
Mach number distribution in the nozzle for variant of 2000 Pa with the roughness configuration: No roughness (**a**) and Ra = 6.3 (**b**).

Figure 31 illustrates the Mach number distribution. For the given configuration, no significant differences are discernible in the depicted Mach number distribution. Due to the subtle variations, these observations were more readily apparent in the graphical representation presented in Figure 28. In contrast to the preceding configurations, the current variant lacks the small regions of elevated flow velocity that extended along the axis towards the diaphragm in the former cases.

**Figure 32:**
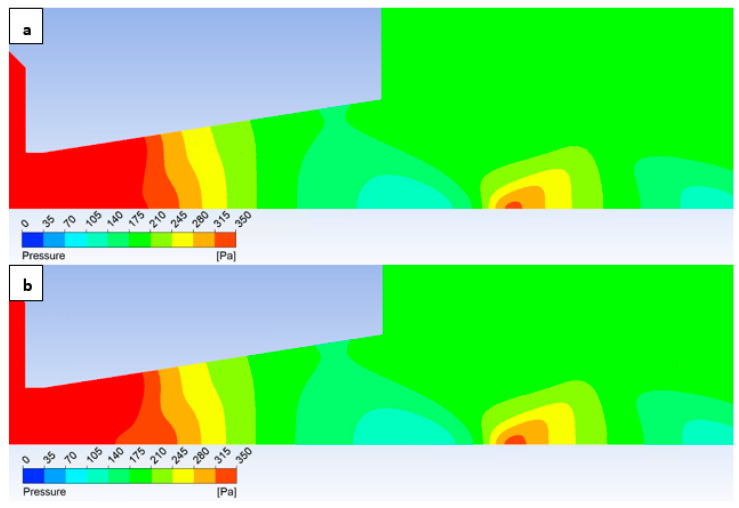
Pressure distribution in the nozzle for variant of 2000 Pa with the roughness configuration: No roughness (**a**) and Ra = 6.3 (**b**).

Consequently, even in the static pressure distribution (Figure 32), significant differences are not readily apparent upon visual inspection. These rather necessitate analysis within the graphs (Figure 29).

**Figure 33:**
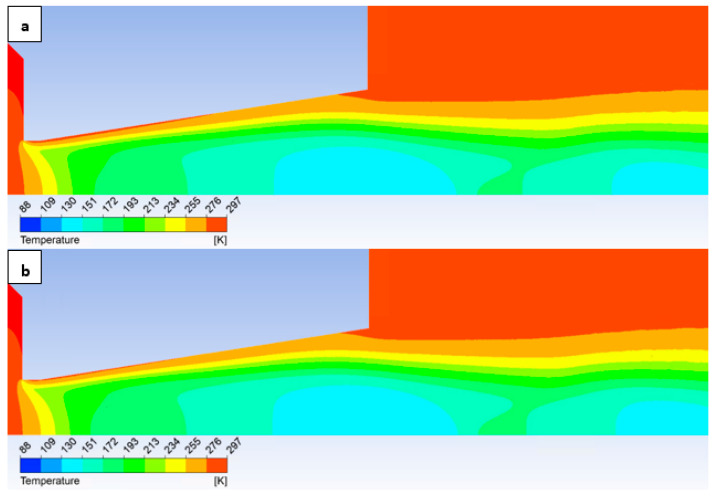
Temperature distribution in the nozzle for variant of 2000 Pa with the roughness configuration: No roughness (**a**) and Ra = 6.3 (**b**).

To ensure comprehensive information, the temperature distribution is once more visually represented in Figure 33.

**Figure 34:**
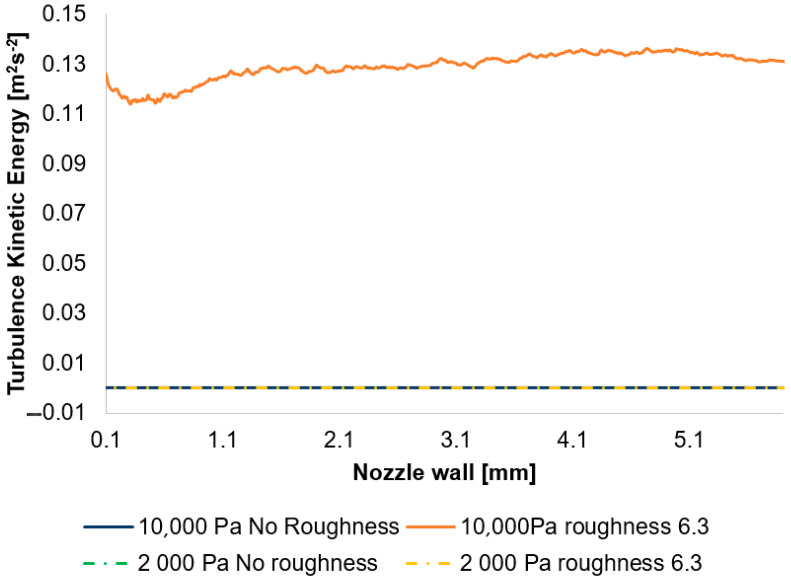
A comparison of turbulence kinetic energy layouts for the 10,000 Pa and 2000 Pa variants with the nozzle wall roughness set in the flow axis.

As previously stated, the discrepancy observed in the 2000 Pa variants is no longer as pronounced as that in the 10,000 Pa variants. This distinction can be illustrated by examining the distribution of turbulent kinetic energy (TKE) evaluated at the nozzle wall (Figure 34).

TKE indicates the intensity of turbulent velocity fluctuations. For this analysis, it is possible to focus on the following influences:

- Intensity of Mixing in the Boundary Layer: A higher TKE value denotes a more efficient mixing process within the boundary layer, which, in turn, affects both the velocity profile and other inherent characteristics of the boundary layer.
- Within the turbulent boundary layer, shear stress continuously generates TKE, which can subtly influence the stability and evolution of the boundary layer.
- The TKE influences effective viscosity, thereby affecting both the thickness and the shape of the boundary layer’s velocity profile.

In this instance, the discrepancy among individual variants is negligible; only in the case of the 10,000 Pa roughness of the 6.3 variant is it notably elevated (Table 7).

An exceptionally low to near-zero TKE signifies laminar flow, a condition arising in this context from a highly specific continuum mechanics regime where inertial forces are substantially reduced, concurrently with a strong dominance of viscous forces.

A significant outcome of the CFD simulations is the observation that for the 2000 Pa variant, an increase in roughness exhibits only a minimal influence, whereas for the 10,000 Pa variant, elevated roughness leads to a heightened intensity of turbulence at the nozzle wall. This effect is attributable to the interaction between the boundary layer and the “core” flow.

The influence on the axial flow velocity is significantly governed by the boundary layer thickness. An elevated value of TKE and its dissipation at the wall indicate more intense mixing within the boundary layer, leading to its growth. A pivotal role in this phenomenon is played by the displacement thickness. This parameter quantifies the effective thickness of a solid wall that would need to be added to the nozzle to compensate for the decelerating effect of the boundary layer on the effective flow area. Consequently, a greater TKE results in a thickening of the boundary layer, thereby reducing the effective flow area. If the effective flow area diminishes due to a thicker boundary layer, the core flow within the nozzle must accelerate more intensely than would be the case in an ideal (inviscid) flow with the given geometry. For a constant mass flow rate, a reduction in the effective cross-section necessarily leads to an increase in velocity.

The values of pressure and temperature are directly dependent on this phenomenon, exhibiting a corresponding decrease. Crucially, wall turbulence impacts the core flow primarily through the modification of the boundary layer thickness and total pressure losses, rather than by direct heat or momentum transfer from turbulent eddies at the wall to the center of the flow.

This research into the distinct characteristics of supersonic flow under the operational pressures of an Environmental Scanning Electron Microscope (ESEM), which are distinguished by a significantly different ratio of inertial to viscous forces, and the specific influence of wall roughness on the nozzle, is of paramount importance. Advances in the development of Advanced ESEM (A-ESEM) operating with higher sample chamber pressures (up to 5000 Pa, exceeding conventional values) specifically necessitate this research to analyze the potential utility of roughness for reducing scattering, which will enable a further enhancement of image sharpness.

### 3.4. Evaluation of Electron Dispersion for Each Variant

The findings discussed above are applied to the aforementioned domain of ESEM research. The pressure gradient exerts a profound impact on the electron beam dispersion as it traverses the differentially pumped chamber. Within the ESEM, a portion of the primary electron beam retains its original trajectory subsequent to its passage through the gaseous medium. These unscattered electrons are then capable of interacting with the specimen and generating a signal, analogous to that of a conventional electron microscope.

During the primary electron passage beam through a gaseous medium at elevated pressures, electrons may undergo collisions with atoms and molecules, resulting in energy loss and directional deflection. The extent of deviation from the initial trajectory is contingent upon the number of collisions (*M_k_*). When *M_k_* is low, the electron’s path remains comparatively undisturbed. Under such conditions, the path length of the electron can be approximated as equivalent to the thickness (d) of the gas layer traversed. The mean number of collisions per electron can be determined using Equation (20) [36].

(20) $$M_{k}=\frac{\sigma_{T}Pd}{kT}$$

where *σ_T_* is the total gas gripping cross-section, *P* is static pressure, *d* is the thickness of the gas layer through which the electron passes, *k* is the Boltzmann constant, and *T* is the absolute temperature.

**Figure 35:**
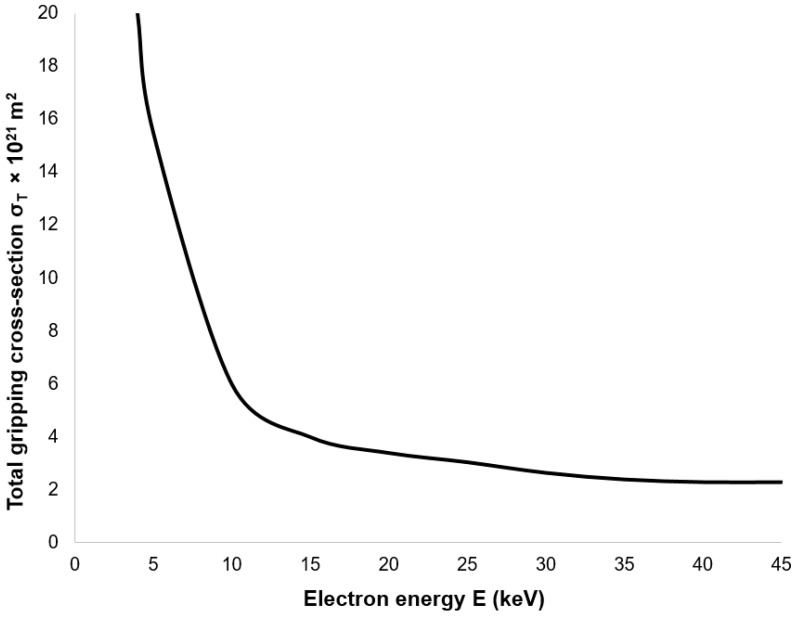
Total gas gripping cross-section as a function of electron energy.

The magnitude of the total gas gripping cross-section decreases with increasing primary electron energy, as illustrated in Figure 35. From a collision perspective, it is therefore advisable to select the primary electron energy just beyond the “bend” in this characteristic, thereby minimizing the scattering of the primary electron beam.

Consequently, the value of *M_k_* dictates the beam dispersion as follows: if *M_k_* < 0.05, the beam dispersion is minimal, not exceeding 5%; if *M_k_* is within the interval [0.05, 3], partial dispersion occurs, ranging from 5% to 95%; and if *M_k_* > 3, complete dispersion is observed, exceeding 95%. The gripping cross-section, denoted as *σ_T_*, represents the close proximity area of a gas particle. A collision eventuates if the electron occupies this spatial location during its transit. Therefore, the gas gripping cross-section is contingent not solely upon the gas species but also upon the accelerating voltage. For the present study, nitrogen was selected in conjunction with an accelerating voltage of 10 keV. Under these conditions, the gripping cross-section *σ_T_* is given as 2 × 10^21^ m^2^, according to reference. Figure 16 illustrates the path traversed by the primary electron beam through the nozzle in a practical ESEM. The nozzle has a length of 5.941 mm. The primary electron beam propagates counter to the direction of the flow [37].

In practice, the trajectory of the primary beam through the differentially pumped chamber in an ESEM is typically 6–8 mm in length, and the flow dynamics are influenced by this confined spatial extent. These findings will serve as a foundation for subsequent analysis under ESEM conditions.

**Figure 36:**
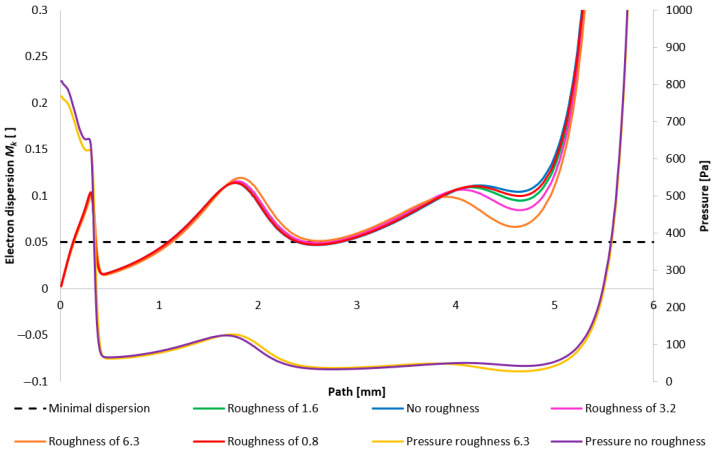
Impact of nozzle wall roughness on the primary beam dispersion for the variant of 5000 Pa.

Figure 36 illustrates the resultant impact of nozzle wall roughness on the primary beam dispersion for the variant of 5000 Pa. The evaluation of the primary electron beam scattering was performed directly on a model constructed according to the actual dimensions of a real ESEM.

**Figure 37:**
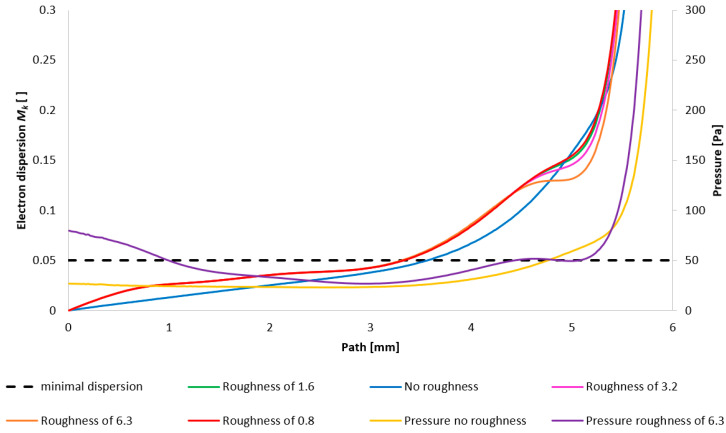
Impact of nozzle wall roughness on the primary beam dispersion for the variant of 1000 Pa.

Figure 37 then depicts the configuration with the lower pressure (1000 Pa variant). Evident in this representation is the phenomenon inferred from prior analyses: that the influence of roughness on the flow field diminishes with decreasing pressure. Consequently, the impact of roughness is less pronounced here compared to the 5000 Pa configuration. It is pertinent to note that this analysis solely addresses the effect of the flow field on beam scattering. Beam dispersion is influenced by a multitude of factors, one of which is the surface roughness itself, exerting a detrimental effect. Therefore, a comprehensive perspective on the entire problem is necessitated.

This research broadly addresses differential pumping conditions within Environmental Scanning Electron Microscopes (ESEMs), with this particular article specifically extending its scope to novel investigations in Advanced Environmental Scanning Electron Microscopy (AESEM). This direction of research is significantly advanced by Vilém Neděla from the Institute of Scientific Instruments of the Czech Academy of Sciences (UPT AVČR), through the development of novel observation methods, including detectors that facilitate ESEM operation at pressures higher than currently conventional. Consequently, this research concurrently investigates the potential for reducing electron beam scattering by roughening the nozzle surface. This approach is predicated on the phenomenon that, at these low pressures, the boundary layer will thicken, leading to a reduction in the effective flow cross-section. A critical consideration, however, is that due to the high ratio of viscous to inertial forces, significant boundary layer separation is averted. This methodology thus yields an improvement in the velocity and pressure conditions that are conducive to a reduction in electron beam dispersion.

## 4. Conclusions

The present paper investigated supersonic flow within a nozzle under low-pressure conditions at the continuum regime boundary. This was achieved using a newly designed experimental chamber that simulates the differential pumping stage in an ESEM. The chamber is configured for the acquisition of pressure and temperature data at the nozzle walls, along the flow axis, and at other strategic locations. This research forms a component of a broader investigation within the field of Environmental Electron Microscopy, which is inherently a multidisciplinary area of study.

The Ansys Fluent system has been successfully tuned, demonstrating a high degree of agreement between experimental results and CFD analyses. Utilizing this validated system, further analyses were conducted to evaluate the influence of surface roughness on the flow characteristics, which affects electron beam scattering in the ESEM.

The influence of aperture and nozzle surface roughness on the pressure distribution along the nozzle wall has been demonstrated. Increased surface roughness resulted in a slight decrease in wall pressure in the vicinity of the oblique shock wave and boundary layer interaction due to minor boundary layer separation. This phenomenon subtly affected the flow characteristics along the axial direction, where the variant with the highest roughness exhibited the highest velocity and lowest pressure. Conversely, for the very low-pressure variant, the impact of wall roughness was less pronounced. As the pressure value decreases, this influence diminishes.

## Figures and Tables

**Table 1:** Used sensor types.

Measured Points	Sensor Type	Range	Accuracy
AV1	Absolute pressure sensor Pfeiffer CMR 361	110 kPa	±0.2%
AV2	Absolute pressure sensor Pfeiffer CMR 362	11 kPa	±0.2%
Z–1	Differential pressure sensor DPS 300	100 kPa	±1% FSO BFSL
1–2	Differential pressure sensor DPS 300	25 kPa	±1% FSO BFSL
2–3	Differential pressure sensor DPS 300	4 kPa	±1% FSO BFSL
3–4	Differential pressure sensor DPS 300	400 Pa	±1% FSO BFSL
4–5	Differential pressure sensor DPS 300	160 Pa	±1% FSO BFSL
5–6	Differential pressure sensor DPS 300	160 Pa	±1% FSO BFSL
6–A	Differential pressure sensor DPS 300	160 Pa	±1% FSO BFSL
A–K	Differential pressure sensor DPS 300	160 Pa	±1% FSO BFSL

**Table 2:** The results of the experimental pressure measurements within the experimental chamber.

	Absolute Pressure in Measured Points [Pa]						
Abs. Pressure in V1 [Pa]	1	2	3	4	5	6	Abs. Pressure in V2 [Pa]
1000	324.4	237.4	193.4	170.4	167.4	169.4	110.4
1500	450	311	241	205	190	190	154
2000	580	389	287	238	213	213	195
2500	697	460	336	276	243	244	237
3000	818	535	383	311	271	275	278
3500	945	616	437	354	309	316	319
4000	1058	684	483	388	339	348	360
5000	1285	828	569	450	395	415	441
6000	1509	965	655	510	452	486	522
7000	1727	1100	737	567	507	556	601
8000	1949	1236	820	625	566	630	681
9000	2168	1368	900	680	627	713	770
10,000	2386	1499	979	733	685	789	851
12,000	2812	1754	1133	837	800	937	1006
14,000	3247	2012	1289	939	920	1090	1162
16,000	3691	2279	1443	103	1043	1244	1320
18,000	4125	2539	1607	1157	1169	1399	1475
20,000	4823	3053	2014	1264	1294	1564	1641

**Table 3:** Evaluation of the Standard Error of the Mean (SEM) derived from measured pressure differences for the 2000 Pa variant.

	Measured Pressure Difference [Pa]						
	Z-1	12	2-3	3-4	4-5	5-6	6-A
Measuring 1	1421	194	98	47	25	0	22
Measuring 2	1414	187	101	44	26	4	29
Measuring 3	1422	191	99	50	24	−1	19
Measuring 4	1421	189	100	50	24	−1	18
Measuring 5	1416	188	97	45	27	−1	19
Measuring 6	1421	191	99	49	25	1	19
Measuring 7	1424	194	97	54	24	−2	15
Measuring 8	1420	193	96	53	24	−2	18
Mean value	1419.9	190.9	98.4	49	24.9	−0.25	19.9
*σ*	3.2	2.7	1.7	3.5	1.1	2	4.2
SEM	1.13	0.95	0.6	1.24	0.39	0.71	1.48

**Table 4:** A comparison of pressure values from experimental measurements versus values from the Ansys Fluent system.

Variant		Absolute Pressure in Measured Points [Pa]					
		1	2	3	4	5	6
2000 Pa	Experiment [Pa]	576	385	287	238	213	213
	CFD [Pa]	603	396	279	222	186	177
	Rel. error [%]	−4.68	−2.86	2.79	6.72	12.68	16.90
4000 Pa	Experiment [Pa]	1058	684	483	388	339	348
	CFD [Pa]	1094	697	479	365	306	320
	Rel. error [%]	−3.40	−1.90	0.83	5.93	9.73	8.05
6000 Pa	Experiment [Pa]	1509	965	655	510	452	468
	CFD [Pa]	1571	981	662	497	422	497
	Rel. error [%]	−4.11	−1.66	−1.07	2.54	6.64	3.91
10,000 Pa	Experiment [Pa]	2386	1 499	979	733	685	789
	CFD [Pa]	2500	1 520	996	747	659	783
	Rel. error [%]	−4.78	−1.40	−1.74	−1.91	3.80	0.76
14,000 Pa	Experiment [Pa]	3247	2012	1289	939	920	1090
	CFD [Pa]	3400	2055	1309	983	894	1086
	Rel. error [%]	−4.71	−2.14	−1.55	−4.69	2.83	0.37

**Table 5:** A comparison of pressure values obtained from experimental measurements and from the Ansys Fluent system with a defined wall roughness value (variant 10,000 Pa).

Measured Points	1	2	3	4	5	6	Average rel. Error [%]
Experiment [Pa]	2386	1499	979	733	685	789	
No roughness [Pa]	2500	1520	996	747	659	783	
Rel. error [%]	−4.78	−1.40	−1.74	−1.91	3.79	0.76	−0.88
Ra = 1.6 [Pa]	2488	1512	985	743	658	783	
Rel. error [%]	−4.25	−0.87	−0.61	1.36	3.94	0.77	−0.40
Ra = 3.2 [Pa]	2467	1501	976	736	656	784	
Rel. error [%]	−3.39	−0.13	0.31	−0.41	4.43	0.64	0.21
Ra = 6.3 [Pa]	2435	1482	959	717	649	784	
Rel. error [%]	−2.05	1.14	2.04	2.18	5.26	0.64	1.53

**Table 6:** A comparison of pressure values obtained from experimental measurements and from the Ansys Fluent system with a defined wall roughness value (variant 2000 Pa).

Measured Points	1	2	3	4	5	6	Average rel. Error [%]
Experiment [Pa]	576	358	287	238	213	213	
No roughness [Pa]	603	396	279	222	186	177	
Rel. error [%]	−4.69	−2.86	2.79	6.72	12.68	16.90	5.26
Ra = 1.6 [Pa]	600	394	276	218	182	175	
Rel. error [%]	−4.17	−2.34	3.83	8.40	14.56	17.84	6.35
Ra = 3.2 [Pa]	597	393	275	217	182	175	
Rel. error [%]	−3.65	−2.08	4.18	8.82	14.55	17.84	6.61
Ra = 6.3 [Pa]	589	388	272	215	181	175	
Rel. error [%]	−2.26	−0.78	5.23	9.66	15.02	17.84	7.45

**Table 7:** A comparison of TKE values for each variant.

Variant	Approx. Value of TKE [m^2^s^−2^]
2000 Pa No roughness	1 × 10^−14^
2000 Pa Ra = 6.3	8 × 10^−13^
10,000 Pa No roughness	1 × 10^−14^
10,000 Pa Ra = 6.3	0.13

## Data Availability

The data presented in this study are available on request from the corresponding author.
